# Exploring the genetic variability and diversity of pearl millet core collection germplasm for grain nutritional traits improvement

**DOI:** 10.1038/s41598-020-77818-0

**Published:** 2020-12-03

**Authors:** Mahalingam Govindaraj, Kedar N. Rai, Anand Kanatti, Hari D. Upadhyaya, Harshad Shivade, Aluri S. Rao

**Affiliations:** 1 International Crops Research Institute for the Semi-Arid Tropics (ICRISAT), Patancheru, 502 324 Telangana India; 2 University of Georgia, Athens, GA 30605 USA

**Keywords:** Agricultural genetics, Plant breeding, Plant genetics, Quantitative trait

## Abstract

Improving essential nutrient content in staple food crops through biofortification breeding can overcome the micronutrient malnutrition problem. Genetic improvement depends on the availability of genetic variability in the primary gene pool. This study was aimed to ascertain the magnitude of variability in a core germplasm collection of diverse origin and predict pearl millet biofortification prospects for essential micronutrients. Germplasm accessions were evaluated in field trials at ICRISAT, India. The accessions differed significantly for all micronutrients with over two-fold variation for Fe (34–90 mg kg^−1^), Zn (30–74 mg kg^−1^), and Ca (85–249 mg kg^−1^). High estimates of heritability (> 0.81) were observed for Fe, Zn, Ca, P, Mo, and Mg. The lower magnitude of genotype (G) × environment (E) interaction observed for most of the traits implies strong genetic control for grain nutrients. The top-10 accessions for each nutrient and 15 accessions, from five countries for multiple nutrients were identified. For Fe and Zn, 39 accessions, including 15 with multiple nutrients, exceeded the Indian cultivars and 17 of them exceeded the biofortification breeding target for Fe (72 mg kg^−1^). These 39 accessions were grouped into 5 clusters. Most of these nutrients were positively and significantly associated among themselves and with days to 50% flowering and 1000-grain weight (TGW) indicating the possibility of their simultaneous improvement in superior agronomic background. The identified core collection accessions rich in specific and multiple-nutrients would be useful as the key genetic resources for developing biofortified and agronomically superior cultivars.

## Introduction

Assuring all people access to sufficient and healthy food remains one of the world’s greatest challenges. The human body requires more than 22 minerals that can be supplied by an appropriate diet^1^. However, the diets of the majority of populations subsisting on cereals, or inhabiting poor regions often lack several of these essential micronutrients. The micronutrients most frequently lacking in human diets are iron (Fe), zinc (Zn), and iodine (I), although Calcium (Ca), Magnesium (Mg), Copper (Cu), and Selenium (Se), can be deficient in the diets of some populations. Studies estimate that, of the world population, 60–80% are Fe deficient, > 30% are Zn deficient, and about 15% is Se deficient^2,3^. Fe and Zn deficiencies, cause anemia and impaired growth (stunting), respectively. The prevalence of these deficiencies is higher in India and sub-Saharan Africa where cereal-based diets predominate. In India, about 60 million children are underweight, 52% of women and 74% of the children (under 5 years’ age) suffer from Fe deficiency-induced anaemia and approximately 52% of the children below 5 years are Zn deficient (stunted). Diets with higher amounts of Mg are associated with a significantly lower risk of diabetes, possibly because of the important role of Mg in glucose metabolism^4,5^. The rising global cardiovascular disease prevalence and low mineral density in the bone may be linked to lower intakes of Mg^6^. Calcium deficiency resulting from a high intake of cereals^7^. Phosphorus (P) helps to metabolize fats and carbohydrates and its deficiency may cause bone associated diseases. Potassium (K) is critical for insulin secretion from pancreatic cells thus K plays a key role in reducing type-2 diabetes. Manganese (Mn) is essential for the activation of enzymes that are mandatory for the digestion. Mn sufficiency will maintain low blood cholesterol levels^8^. Considering these importance worldwide, micronutrient malnutrition results in an enormous socioeconomic cost to the developing world^9,10^.

Pearl millet (*Pennisetum glaucum* (L.) R.Br.) is a staple cereal crop in terms of production and one of the major sources of food, fodder, and feed in many countries of sub-Saharan Africa and northwestern states in India. Developing nutrient-dense cultivars and their cultivation will help overcome these deficiencies and will be of great value to the global population. The success of crop improvement programs depends on the extent of characterized germplasm for various traits available to breeders for breeding desirable cultivars. Pearl millet core collections representing the diversity of the entire collection facilitates better utilization of germplasm resources and diversity^11^. Like other traits, variability for micronutrients found in core collections can be efficiently utilized upon primary data become available to enhance grain micronutrients in this crop. Although selecting micronutrient-dense lines among existing breeding populations and varieties within breeding programs is the first approach, the potential for micronutrient enhancement through deliberate selection from germplasm collections is much greater than by selection within available breeding lines or varieties^12,13^.

During more than 45 years of pearl millet breeding at ICRISAT, germplasm was largely studied for and sources were identified for biotic and abiotic tolerance and yield component traits. However, very little information and no systematic study is available on grain nutrients in the germplasm collection except for a few studies in identifying variation for Fe and Zn in *Iniadi* germplasm^14^. Considering pearl millet’s role in addressing malnutrition issues in India and Africa, beyond the high Fe and Zn densities, investigating variability for macro- and micronutrients adding another dimension to the genetic improvement of this crop. Therefore, this study aimed to systematically characterize the genetic variability for grain micronutrients in a set of core collection accessions^15^ and to identify promising sources for multiple nutrients as donors for the pearl millet biofortification breeding program.

## Results

### Analysis of variances

ANOVA of the data on 212 accessions revealed highly significant mean squares attributable to genotypes for 50% flowering, TGW, Fe, and Zn in individual seasons as well as in pooled analysis over seasons (Table 1). Similarly, the genotypic mean squares for 39 accessions for 50% flowering, TGW, and all micronutrients, except for K and P in the 2011 rainy season, were highly significant in individual seasons and the pooled analysis over seasons, (Table 2). Although the mean square attributable to G × E interaction was significant for most of the traits their values were lower compared to that attributable to genotypes. The proportion of total variability, as judged by the sum of squares, explained by G × E interaction relative to that of genotype was 17% for Fe, 25% for Zn, 18% for days to 50% flowering, and 24% for TGW in 212 accessions. In the ANOVA involving 39 accessions, the G × E interactions were significant for 50% flowering, TGW, Zn, Cu, Molybdenum (Mo), Ca, Mg, Sodium (Na), and non-significant for Fe, Mn, Nickel (Ni), K and Sulphur (S). The proportion of total variability explained by G × E interaction relative to that of genotype was low to moderate for all the traits (10–32%) except Cu (45%) and Mg (49%). The Bartlett's test indicated homogeneity of error variances for most traits except for Ni, K, and P.

**Table 1:** Mean squares for iron (Fe), zinc (Zn), days to 50% flowering and 1000-grain weight of a set of 212 pearl millet core collection accessions evaluated during the 2011 summer and 2011 rainy seasons, Patancheru, India.

Source of variation	Degrees of freedom	Mean square											
		Fe			Zn			Days to 50% flowering			1000-grain weight		
		Pooled	S	R	Pooled	S	R	Pooled	S	R	Pooled	S	R
Environments (E)	1	5494**			9105**			1657**			173**		
Replication/E	2 (1)	239**	429**	48*	144**	150**	139**	54**	63**	46**	19**	4*	34**
Genotypes (G)	211	550**	318**	327**	262**	150**	178**	129**	81**	70**	19**	10**	14**
G × E	211	95**			67**			23**			5**		
Residuals	422 (211)	13	13	12	16	14	18	7	7.2	6.6	0.9	0.9	0.9
Mean		54	57	52	49	52	45	56	57	54	9	9	10
SE ±		4	4	3	4	4	4	3	3	3	1	1	1
LSD (5%)		5	7.2	4.9	5.6	7.5	6	3.7	5.3	3.6	1.3	1.8	1.3
CV (%)		6.6	6.5	6.8	8.3	7.3	9.5	4.7	4.7	4.8	10.1	10.5	9.7
Homogeneity of error variance		NS	–	–	NS	–	–	NS	–	–	NS	–	–

Values in the parenthesis are degrees of freedom for individual environments.

*S* summer, *R* rainy, *NS* non-significant.

*,** Significant at the 0.05 and 0.01 probability levels, respectively.

**Table 2:** Mean Squares for days to 50% flowering, 1000-grain weight (TGW) and nutritional traits of 39 of 212 core collection accessions evaluated during the 2011 summer and 2011 rainy seasons, Patancheru, India.

Trait	Environment (E)		Rep	Genotype (G)	G × E	Error	Mean	SE	CV (%)	LSD	Bartlett test
	df	1	2 (1)	38	38	76(38)					
Days to 50% flowering	Pooled	57.28**	26.82*	158.87**	15.2*	9.67	53	1.6	2.3	4.4	SIG
	S		53.64*	87.68**		13.14	54	2.6	3	7.3	
	R		0.1	86.96**		6.11	52	1.7	2.5	5	
TGW	Pooled	2.68	2.07	21.58**	2.81*	1.54	11	0.6	7.1	1.7	SIG
	S		0.48	11.08**		2.02	11	1	9.1	2.9	
	R		3.66	14.28**		1.02	11	0.7	7.5	2	
Fe	Pooled	541.61**	104.28	343.88**	61.44	43.09	70	3.3	2.6	9.2	NS
	S		207.00*	217.52**		32.59	72	4	2.8	11.6	
	R		1.56	202.78**		55.79	68	5.3	3.4	15.1	
Zn	Pooled	125.15*	5.33	195.46**	52.01*	31.54	52	2.8	3.2	7.9	NS
	S		5.51	142.66**		23.22	53	3.4	3.5	9.8	
	R		5.15	109.36**		41.83	51	4.6	4.2	13.1	
Mn	Pooled	0.34	12.86*	12.40**	3.12	3.48	14	0.9	7	2.6	NS
	S		2.16	6.13*		3.39	14	1.3	8.3	3.7	
	R		23.56*	9.02*		3.61	14	1.3	8.4	3.8	
Cu	Pooled	25.79**	0.48	2.26**	1.02*	0.66	5.7	0.4	11.1	1.1	NS
	S		0.96	1.91**		0.56	6.2	0.5	11.7	1.5	
	R		0	1.56*		0.84	5.2	0.6	15.6	1.9	
Mo	Pooled	0.31**	0.17*	0.42**	0.08**	0.04	1.4	0.1	23.2	0.3	NS
	S		0.18*	0.28**		0.03	1.3	0.1	28.3	0.4	
	R		0.17	0.26**		0.05	1.4	0.2	26.9	0.4	
Ni	Pooled	1.58**	0.07	0.20**	0.05	0.05	1.2	0.1	28.2	0.3	SIG
	S		0.1	0.15*		0.07	1.3	0.2	32.3	0.5	
	R		0.04	0.11**		0.03	1	0.1	33.6	0.3	
Ca	Pooled	66,255**	1647*	4040**	1001**	455	173	10.7	1.9	30	NS
	S		2852*	3087**		433	199	14.7	1.9	42.1	
	R		443	2054**		495	143	15.7	2.8	45	
Mg	Pooled	469,383**	71,230*	68,932**	33,490**	15,150	1333	61.5	0.6	173.3	NS
	S		8556	70,676**		13,305	1385	81.6	0.7	233.5	
	R		133,903*	42,238*		18,963	1266	97.4	0.8	278.8	
Na	Pooled	20.95*	5.65	23.85**	7.54*	4.85	14	1.1	7.5	3.1	NS
	S		2.35	19.69**		4.57	13	1.5	9.1	4.3	
	R		8.95	13.78**		5.11	14	1.6	8.8	4.6	
K	Pooled	3,955,015**	417,150	702,626**	208,042	296,831	4164	272.4	0.4	767.3	SIG
	S		7424	516,745**		174,612	4339	295.5	0.4	845.9	
	R		826,875	436,758^NS^		466,875	3963	483.2	0.6	1383.2	
P	Pooled	7,002,492**	114,572	475,523**	176,710	125,801	3634	177.3	0.4	499.5	SIG
	S		113,906	377,698**		62,939	3880	177.4	0.3	507.9	
	R		115,238	298,483^NS^		223,238	3342	334.1	0.5	956.5	
S	Pooled	65,926*	52,506*	66,357**	16,046	13,266	1362	57.6	0.6	162.2	NS
	S		22,756	56,481**		14,558	1382	85.3	0.7	244.3	
	R		82,256	30,641**		11,853	1341	77	0.7	220.4	

*S* summer, *R* rainy, *SIG* significant at the 0.05 probability, *NS* non-significant. Values in the parenthesis are degrees of freedom (df) for individual environment.

*,**Significant at the 0.05, 0.01 probability levels, respectively.

### Nutrient content in field soil

The mean available Fe and Zn contents in the experiential fields in the first 30 cm soil were 14.3 mg kg^−1^ and 2.5 mg kg^−1^, respectively in the 2011 summer season and 14.7 mg kg^−1^ Fe and 9.9 mg kg^−1^ Zn in the 2011 rainy season.

### Genetic variability and heritability

Among the 212 core collection accessions, days to 50% flowering ranged from 41 to 70 days in summer and from 39 to 72 days in the rainy season (Table 3). The overall mean over the seasons was 56 days, and 97 accessions flowered early and took 41–55 days to 50% flowering. Based on a two-season evaluation of 212 accessions, TGW varied from 5 to 15 g and 91 accessions had higher TGW than the trial mean (9 g). The Fe content based on XRF estimate varied from 34 to 90 mg kg^−1^ and 102 accessions showed higher Fe than the trial mean (71 mg kg^−1^). The Zn estimated using XRF varied from 30 to 74 mg kg^−1^ and 96 accessions showed a greater Zn content than the trial mean (49 mg kg^−1^). High estimates of heritability in the broad sense were observed for all traits, 84% for Fe, 77% for Zn, 83% for days to 50% flowering, and 78% for TGW.

**Table 3:** Range, mean, and coefficient of variation (CV%) for days to 50% flowering, 100-grain weight (TGW) and nutritional traits in a set of 212 and 39 pearl millet core collection accessions evaluated during the 2011 summer and 2011 rainy seasons, Patancheru, India.

Trait	Range		Mean		No. of entries with greater trial mean		CV %	
	(39)	(212)	(39)	(212)	(39)	(212)	(39)	(212)
Days to 50% flower (d)	41–67	41–70	53.3	56	18	112	2.3	4.7
TGW (g)	6–16	5–15	11.1	9	17	91	7.1	10.1
**(mg kg**^**−1**^)								
Fe	48–93	34–90	70.7	54	19	102	2.6	6.6
Zn	33–65	30–74	52.6	49	23	96	3.2	8.3
Mn	10–17	–	13.8	–	19	–	7.0	–
Cu	4–7	–	5.7	–	21	–	11.1	–
Mo	0.6–2.2	–	1.4	–	18	–	23.2	–
Ni	0.7–1.9	–	1.2	–	19	–	28.2	–
Ca	85–249	–	172.6	–	19	–	1.9	–
Mg	1127–1837	–	1335.1	–	15	–	0.6	–
Na	8–22	–	14.0	–	18	–	7.5	–
K	3167–5133	–	4174.4	–	20	–	0.4	–
P	2900–4733	–	3644.7	–	17	–	0.4	–
S	1123–1703	–	1366.9	–	16	–	0.6	–

Over two seasons evaluation of the selected 39 accessions, days to 50% flowering varied from 41 to 67 days, and 18 accessions flowered earlier than the trial mean (53 days) (Table 3). TGW varied from 6 to 16 g with 17 accessions had grain with larger TGW than the trial mean (11 g). The wet lab micronutrient analysis using ICP-OES showed a greater variability for micronutrient contents. However, higher variability was observed for Ca (85–249 mg kg^−1^), Fe (48–93 mg kg^−1^), Zn (33–65 mg kg^−1^) and P (2900–4733 mg kg^−1^). Nineteen accessions each for Ca and Fe, 23 accessions for Zn, and 17 accessions for P exceeded the trial mean of respective nutrients. Mn content varied from 10 to 17 mg kg^−1^ and 19 accessions exceeded the trial mean (13.8 mg kg^−1^). Variation for Cu was 4–7 mg kg^−1^ and 21 accessions surpassed the trial mean (5.7 mg kg^−1^). Mo varied from 0.6 to 2.2 mg kg^−1^ and 18 accessions had higher Mo than the trial mean (1.4 mg kg^−1^). Mg varied from 1127 to 1837 mg kg^−1^ and 15 accessions had higher Mg than the trial mean (1335 mg kg^−1^). Na varied from 8 to 22 mg kg^−1^ and 18 accessions had higher Na than the trial mean (14 mg kg^−1^). K varied from 3167 to 5133 mg kg^−1^ with 20 accessions exceeded trial mean (4174 mg kg^−1^). S varied from 1123 to 1703 mg kg^−1^ with 16 accessions exceeded trial mean (1367 mg kg^−1^). Eighteen accessions had higher Fe, 87 accessions had higher Zn than the controls (ICTP 8203, 70 mg kg^−1^ Fe and 49 mg kg^−1^ Zn) whereas 35, 5, 4, and 6 accessions exceeded the control, respectively, for Na, Mg, P, K. High heritability was observed for all the traits (data not presented). Heritability varied from moderately high (57%) to very high (91%). Very high heritability traits were days to 50% flowering (91%), TGW (87%), Fe (83%), Mo (81%), Ca (77%), S (76%), Zn (75%), Ni (75%), Mn (74%), Na (70%), K (68%) and P (65%).

### Nutrient-dense germplasm

To identify multiple nutrient germplasm data on 39 core collection accessions (a subset of 212 accessions) that had data on all micronutrients with agronomic traits were considered. The top 10 accessions having superior levels of each nutrient trait were selected except for Ni and Na, for which the accessions having the lowest levels were selected as per desirability (Table 4). All these high nutreint-dense accessions flowered in 45–67 days and had 6–16 g TGW. The low Ni and Na accessions also had a similar range of flowering (45–67 days) and TGW (7–16 g). For instance, the top 10 nutrient specific accessions had 78–93 mg kg^−1^ Fe content, 58–65 mg kg^−1^ Zn content, and 201–249 mg kg^−1^ Ca content. Of the 10 high-Fe accessions, only one accession had > 58 mg kg^−1^ Zn and 4 accessions recorded high-Ca content (201–235 mg kg^−1^). This study also found the best accessions that had higher levels of multiple nutrients. The top 15 accessions were identified considering high values for all the individual nutrients except for Ni and Na where the lowest 15 accessions were taken into consideration (Table 5). These 15 accessions originated from five countries. The accessions namely IP 9351, IP 11784, IP 12507, IP 9407, IP 3329, IP 3749, IP 7536, IP 5316, IP 4454, IP 9496, IP 14148, IP 12939, IP 11316, IP 7208 and IP 9572 had the best combinations of multiple nutrients. Of these 15, three accessions (IP 9351 IP 9407, and IP 3329) were the best source for 11 nutrients while IP 11784 was a good source for 10 nutrients. Similarly, IP 12507 and IP 12939 were identified for the high value of 9 nutrients. Of these top 15 accessions, 10 accessions had a greater Fe (> 72 mg kg^−1^) and Zn (> 55 mg kg^−1^) content while these had moderate Ca content (161–239 mg kg^−1^). The top 15 accessions had a wider range of days to 50% flowering from 42 to 64 days and TGW ranging from low (7 g) to high (16 g).

**Table 4:** Top 10 core collection accessions with high value for each nutritional trait and their agronomic performance based on evaluation during the 2011 summer and 2011 rainy seasons, Patancheru, India.

Trait	Class	Top 10 accessions (IP#) with high trait value (mg kg^−1^)	Range	
			Days to 50% flowering (d)	1000-grain weight (g)
Fe	High	3329, 7536, 8972, 9301, 10,394, 12,682, 15,817, 17,620, 17,690, 17,707	46–60	7–14
Zn	High	3329, 3749, 4454, 10,471, 11,584, 11,784, 12,181, 13,900, 15,614, 17,217	51–63	6–12
Mn	High	7536, 8972, 9572, 10,394, 13,384, 15,402, 15,614, 17,217, 17,620, 17,707	46–64	6–14
Cu	High	3329, 3626, 7208, 9351, 10,394, 12,507, 12,682, 13,384, 13,900, 15,614	46–60	6–14
Mo	High	3329, 13,900, 15,614, 3749, 10,471, 11,316, 11,353, 11,584, 11,784, 17,217	51–67	6–16
Ni	Low*	11,316, 12,682, 13,384, 7536, 11,320, 9407, 11,784, 14,148, 9416, 10,471	45–61	7–16
Ca	High	15,402, 9416, 17,707, 9351, 15,614, 17,620, 11,584, 14,148, 7536, 5316	45–60	6–14
Mg	High	15,614, 9572, 7208, 17,217, 7536, 3626, 3329, 4454, 12,507, 10,471	51–64	6–10
Na	Low*	5316, 13,384, 3626, 12,181, 17,690, 7838, 4454, 11,353, 10,471, 12,682	52–67	8–14
K	High	9572, 10,471, 12,682, 11,584, 3329, 12,507, 14,148, 15,614, 11,316, 11,353	51–67	6–16
P	High	9572, 15,614, 3329, 7536, 12,507, 14,148, 7208, 10,471, 12,682, 17,217	51–64	6–11
S	High	9572, 17,217, 12,507, 7536, 7838, 3329, 4454, 15,614, 3626, 11,784	51–64	6–10

*Low quantity of Ni and Na is desirable.

**Table 5:** Core collection accessions with high values of multiple nutritional traits s based on evaluations in the 2011 summer and 2011 rainy seasons, Patancheru, India.

Accession	Country	Cluster no Figure 1b	Fe	Zn	Mn	Cu	Mo	Ni	Ca	Mg	Na	K	P	S	Days to 50% flowering	1000-grain weight
			mg kg^−1^												(d)	(g)
IP 3329	India	1	**78**	**62**	**14**	**7.3**	**1.9**	1.4	155	**1438**	14	**4800**	**4400**	**1533**	60	7
IP 3749	India	2	**78**	**56**	**17**	**5.9**	1.2	**0.8**	**204**	**1523**	13	4367	**4200**	**1577**	58	9
IP 4454	India	2	**91**	**57**	**16**	3.9	0.7	1.3	**212**	1327	18	3867	3300	1195	56	8
IP 5316	Niger	2	**82**	51	**17**	**6.1**	1.3	1.1	**235**	1280	18	**4400**	3650	**1400**	57	11
IP 7208	India	1	**93**	47	**16**	**5.9**	1	1.9	**180**	**1377**	16	**4433**	3700	1303	53	7
IP 7536	India	1	**82**	**57**	10	**7.2**	**1.5**	**0.7**	119	1317	13	**4900**	**4033**	1237	58	7
IP 9351	Ghana	4	71	**61**	**17**	**7.5**	**1.8**	1.1	**229**	**1837**	22	**4633**	**4500**	**1523**	46	14
IP 9407	Ghana	4	64	**59**	**16**	**5.6**	**2.2**	1.3	**179**	**1613**	14	**4400**	**4025**	**1638**	45	13
IP 9496	Ghana	4	**76**	**65**	**14**	5.5	**2.2**	1.4	**208**	1333	16	**4825**	3650	1353	42	15
IP 9572	Ghana	1	**72**	**61**	**12**	5.4	**1.8**	**0.9**	105	**1390**	12	**4967**	**4033**	**1455**	64	10
IP 11316	Burkina Faso	5	70	**58**	12	5.1	**1.5**	1.4	85	**1420**	**11**	3375	3400	**1530**	56	16
IP 11784	India	2	52	46	**16**	**6.1**	**1.3**	1.5	**164**	**1740**	20	**5133**	**4733**	**1703**	51	10
IP 12507	India	1	**76**	42	12	**6.7**	**1.5**	1.2	128	**1405**	15	**4775**	**4100**	**1583**	51	10
IP 12939	Ghana	4	**74**	**56**	11	**6.6**	**1.5**	1.2	**161**	**1453**	**10**	3767	**3800**	**1480**	43	13
IP 14148	Zimbabwe	5	69	55	12	5.3	1.2	**0.9**	**205**	1307	13	**4667**	**4100**	1298	59	10
Minimum value		52	42	10	4	1	1	85	1280	10	3375	3300	1195	42	7	
Maximum value		93	65	17	7	2	2	235	1837	22	5133	4733	1703	64	16	

Values in bold indicate higher levels of multiple nutrients except for Ni and Na where the lowest is desirable.

### Cluster analysis

**Figure 1:**
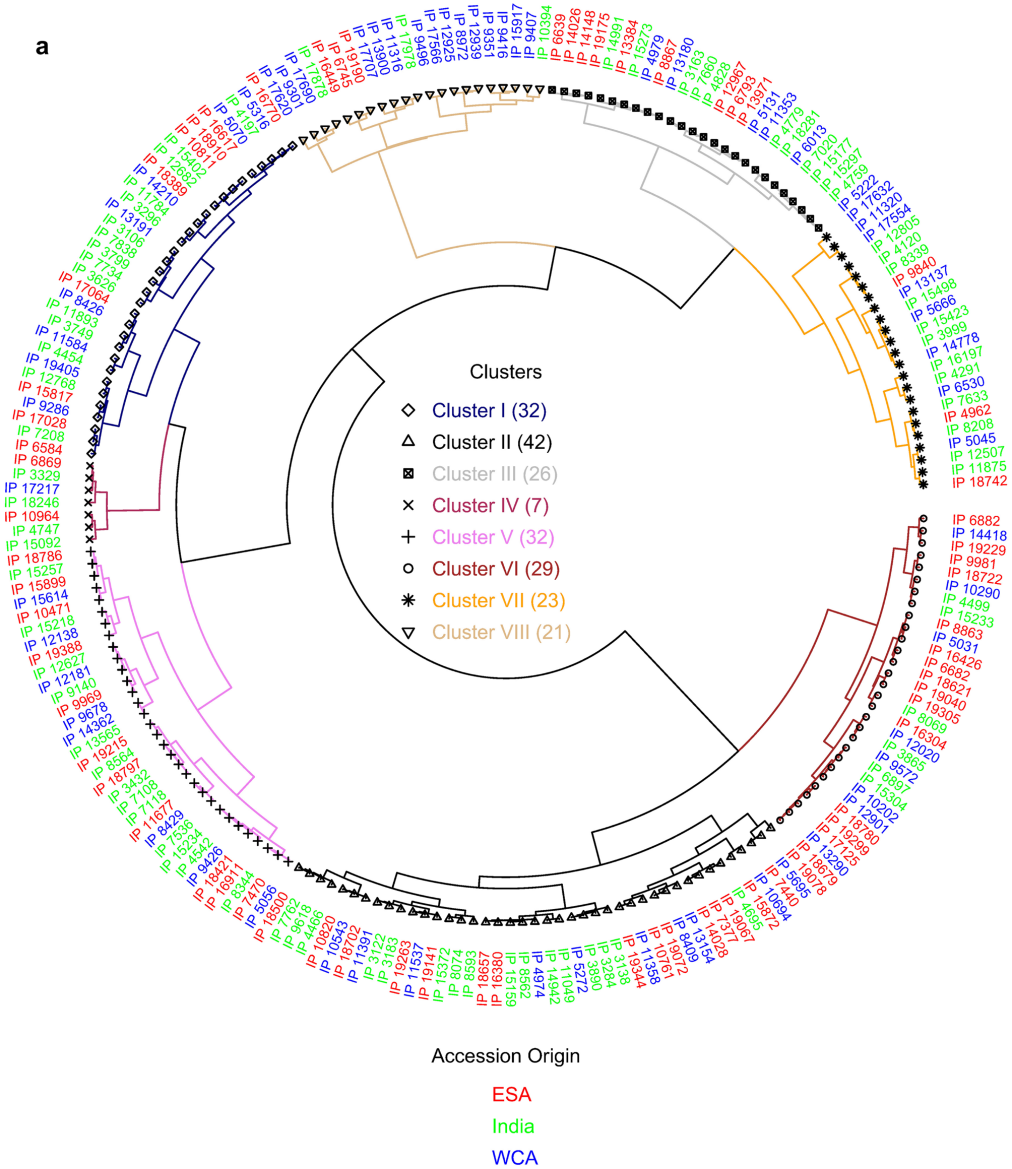

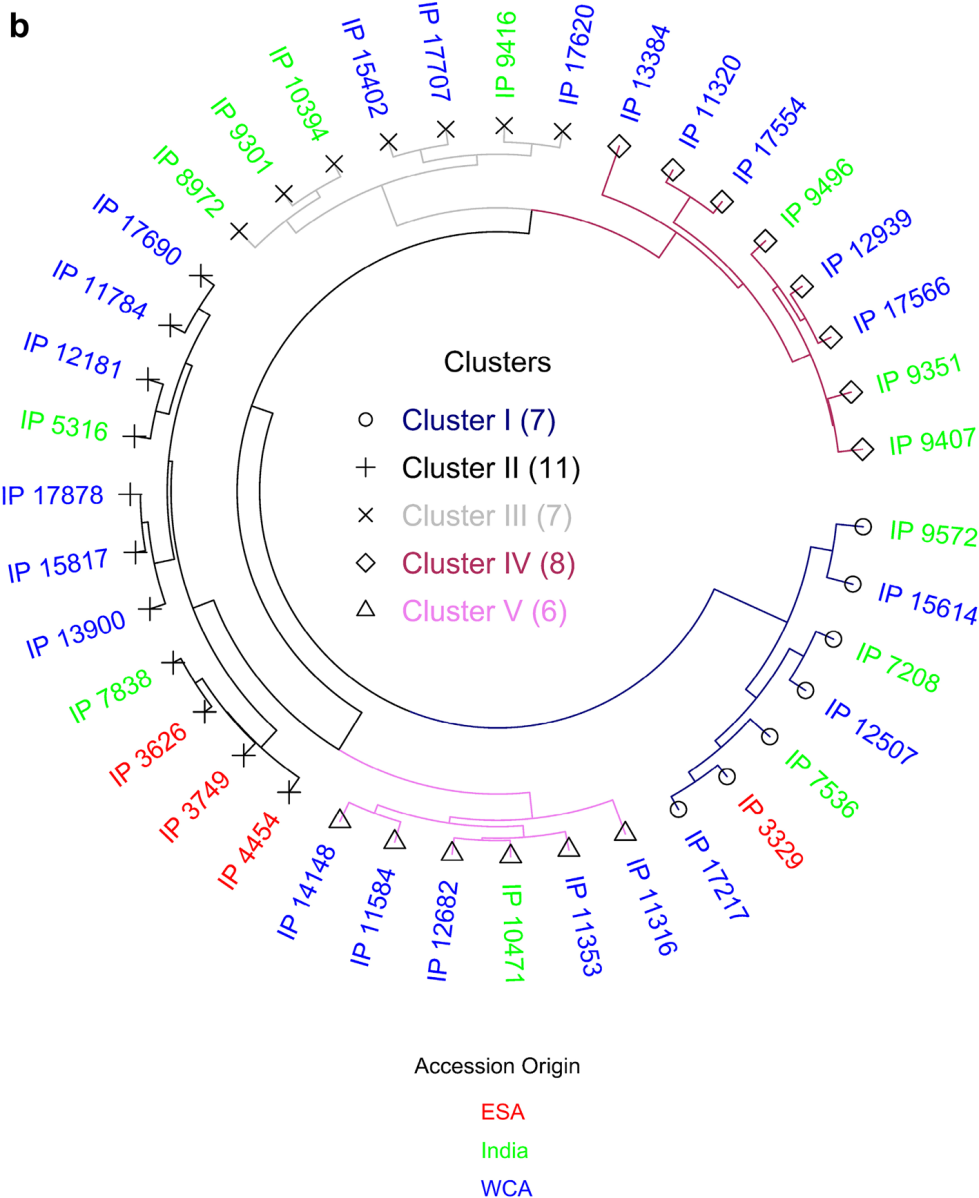
Cluster diversity showing the pairwise relatedness of (**a**) 212 pearl millet core collection accessions based on grain Fe, grain Zn, and agronomic traits, (**b**) selected 39 pearl millet core collection accessions based on 12-grain minerals and agronomic traits.

All 212 core accessions were grouped into 8 distinct clusters (Supplementary Table S4, Fig. 1a). The number of accessions in each cluster varied from 7 (in cluster 4) to 42 (in cluster-2). Cluster1 and cluster 5 consisted of 32 accessions each; cluster-3 consisted of 26 accessions, while 29, 23, and 21 accessions were grouped into cluster 6, cluster 7, and cluster 8, respectively. The mean values of each cluster were significantly different for all the traits. Clusters 1, 4, and 8 had higher mean value for both Fe (65–72 mg kg^−1^) and Zn (52–68 mg kg^−1^) densities. While cluster 2 and cluster 6 had accessions, which had lower cluster mean values for Fe (42–45 mg kg^−1^) and low to moderate Zn (35–58 mg kg^−1^). Interestingly, all the accessions in cluster 8 also had higher TGW (> 10 g, i.e. bold grains) and 67% of entries (14 accessions) in this cluster had early to medium days to 50% flowering (41–50 days). Similarly, cluster 1 contained 44% of entries (14 accessions) with > 10 g TGW and medium to late flowering (50–66 days); while cluster 4 accessions were recorded with small grains i.e. 7–9 g TGW but coupled with late flowering (> 56–61 days). Accessions in clusters 8, 1, and 4 were found to have more desired traits; these accessions will have contributed to higher Fe and Zn as well as large grains size. Of the 212 accessions, 102 and 90 accessions exceeded the trial mean for Fe and Zn content, respectively, 18 accessions surpassed Fe content of the control cultivar (ICTP 8203, 70 mg kg^−1^ Fe and 49 mg kg^−1^ Zn) for these micronutrients contents and 11 of them were distributed to cluster 8, four in cluster 1, two in cluster 4, and one in cluster 5 (Supplementary Table S4) Cluster 8 had the highest mean value for Fe, of the 21 genotypes in this cluster 18 were from Africa and 3 genotypes, IP 17978, IP 10394, and IP17878 were from India and these had 64, 75, 80 mg kg^−1^ Fe and 55, 47, 55 mg kg^−1^ Zn, respectively. While cluster 4 had higher mean values for Zn and 4 of the 7 genotypes originated from India. Cluster 2 had the highest number of genotypes grouped and genotypes in this cluster had lower values for both Fe and Zn, of the accessions, 16 (38%) were from India, and 26 (62%) from Africa.

Cluster analysis of 39 accessions based on 14 traits, were grouped into five distinct clusters at 94% similarity (Supplementary Table S5 and Fig. 1b). The number of accessions in the clusters varied from 6 accessions in cluster 5, 7 each in cluster1 and 3, 8 in cluster 4, and 11 in cluster 2. Cluster1 had higher cluster mean values for Mg, P, S, Cu, and cluster 2 had lower cluster means for most of the traits including Fe. Cluster3 had higher cluster mean values for Fe, Mn, and Ca, and cluster4 had higher cluster mean for TGW and lower value for days to 50% flower, and cluster5 had a higher cluster mean for Zn, Mg, Mo, and K and the lower mean value for Ni.

### Correlation among nutrients and flowering and grain weight

The estimates of Fe and Zn using XRF and ICP were highly correlated in the study involving 212 and 39 accessions in the 2011 Summer season (r = 0.78–0.85 for Fe; 0.87–0.88 for Zn), in the 2011 Rainy season (r = 0.74–0.0.86 for Fe; 0.85–0.89 for Zn and over both seasons (r = 0.82–0.83 for Fe; 0.0.89–0.92 for Zn). Based on two season pooled data, the correlation analysis was conducted to determine the relationships among grain micronutrients, macronutrients, and with two important agronomic traits (Table 6). Correlation between Fe and Zn was found highly significant and positive (r = 0.43–0.51; *p* < 0.01). Fe content was positively and significantly (*p* < 0.05) correlated with Ca (r = 0.33; *p* < 0.05) and Ni content (r = 0.32; *p* < 0.05). Fe content had non-significant correlations with other micronutrients (r = 0.00 to − 0.18). Zn content was positively and highly significantly (r = 0.36–0.46; *p* < 0.01) correlated with Mo and S while non-significantly mostly positively correlated with other nutrients. A highly significant and positive correlation of Mg with Na, P, K, S, Cu, and Mo was seen. Ca had a largely positive association with all nutrients but was significantly associated only with Fe and Mn. Although P and K had a positive correlation with other nutrients, significant correlations were observed only for P with K, Cu, Mo, and S whereas K was associated with Mg and Na. Correlation between days to 50% flowering and TGW was negative and significant. All the micronutrients recorded positive correlations with 50% flowering and highly positive and significant correlation of days to 50% flowering with Zn, Mo, Mg, K, and P was seen. Fe content did not correlate with days to 50% flower while Zn content recorded positive and significant correlation. On the other hand, the correlation between Fe density and TGW was low negative and non-significant but a negatively significant correlation was observed between Zn density and TGW. In general, TGW showed an undesirable correlation with almost all the micronutrients, and correlation was highly significant with Mo, Mg, P, and S.

**Table 6:** Correlation among grain nutritional and agronomic traits in 39 core collection accessions evaluated in the 2011 summer and 2011 rainy seasons, Patancheru, India.

Crop Season	Trait	Days to 50% flowering	1000-grain weight	Fe	Zn	Mn	Cu	Mo	Ni	Ca	Mg	Na	K	P
S	1000-grain weight	− 0.54**												
R		− 0.52**												
Pooled		− 0.57**												
S	Fe	− 0.06	− 0.23											
R		− 0.01	0.03											
Pooled		− 0.05	− 0.09											
S	Zn	0.36*	− 0.45**	0.43**										
R		0.22	− 0.01	0.51**										
Pooled		0.40*	− 0.33*	0.46**										
S	Mn	0.00	− 0.14	0.22	− 0.15									
R		0.09	− 0.21	0.00	− 0.15									
Pooled		0.04	− 0.19	0.13	− 0.16									
S	Cu	0.09	− 0.38*	0.02	0.02	0.12								
R		0.01	− 0.09	0.24	0.17	0.22								
Pooled		0.10	− 0.35*	0.15	0.08	0.17								
S	Mo	0.44**	− 0.36*	− 0.05	0.47**	− 0.20	0.27							
R		0.38*	− 0.41**	− 0.12	0.30	− 0.01	− 0.02							
Pooled		0.49**	− 0.42**	− 0.11	0.46**	− 0.19	0.16							
S	Ni	− 0.09	− 0.07	0.39*	0.30	0.19	0.14	0.06						
R		0.03	− 0.12	0.41**	− 0.08	0.25	0.03	− 0.06						
Pooled		− 0.05	− 0.15	0.32*	0.07	0.20	0.16	− 0.03						
S	Ca	− 0.11	− 0.07	0.33*	0.33*	0.41**	− 0.01	− 0.16	0.19					
R		− 0.09	0.22	0.22	0.06	0.53**	0.29	− 0.29	− 0.03					
Pooled		− 0.10	0.09	0.33*	0.14	0.47**	0.04	− 0.20	0.11					
S	Mg	0.41**	− 0.67**	0.13	0.34*	0.29	0.44**	0.27	0.28	0.24				
R		0.22	− 0.42**	− 0.18	0.08	0.46**	0.32*	0.26	0.09	0.07				
Pooled		0.53**	− 0.75**	0.02	0.24	0.37*	0.41**	0.39*	0.27	0.03				
S	Na	− 0.05	0.01	0.21	− 0.09	0.23	− 0.02	− 0.06	0.18	0.23	0.27			
R		− 0.04	0.00	− 0.05	− 0.12	0.35*	0.04	0.05	0.32*	0.03	0.42**			
Pooled		− 0.10	0.04	0.11	− 0.07	0.37*	0.01	0.01	0.24	0.20	0.33*			
S	K	0.33*	− 0.15	0.01	− 0.08	0.30	0.21	0.06	0.18	0.23	0.52**	0.33*		
R		0.32*	− 0.15	0.08	0.16	0.20	0.20	0.36*	0.06	− 0.03	0.39*	0.26		
Pooled		0.43**	− 0.16	0.10	0.06	0.08	0.16	0.26	0.05	0.09	0.42**	0.32*		
S	P	0.47**	− 0.61**	0.07	0.32*	0.14	0.46**	0.22	0.16	0.16	0.89**	0.14	0.64**	
R		0.35*	− 0.37*	− 0.05	0.20	0.32*	0.52**	0.27	0.04	0.06	0.75**	0.29	0.70**	
Pooled		0.57**	− 0.62**	0.03	0.24	0.22	0.51**	0.40*	0.11	0.02	0.83**	0.19	0.71**	
S	S	0.43**	− 0.66**	0.04	0.57**	0.02	0.37*	0.48**	0.29	0.15	0.72**	− 0.05	0.17	0.71**
R		0.45**	− 0.55**	− 0.03	0.21	0.31*	0.47**	0.31*	0.10	0.02	0.50**	0.14	0.22	0.51**
Pooled		0.50**	− 0.73**	− 0.01	0.36**	0.16	0.36*	0.44**	0.23	− 0.02	0.72**	− 0.02	0.18	0.66**

*,**Significant at 0.05, 0.01 probability levels, respectively; S, Summer; R, Rainy.

## Discussion

A large proportion of the global population suffers from micronutrient deficiency. Among the micronutrients, Fe (60–80% population), Zn (30%), and Se (15%) deficiency is widespread^2,3^. The social costs of micronutrient deficiency are devastating to the countries as it results in anaemia (Fe deficiency) and impaired growth (Zn deficiency). Lower intake of other micronutrients such as Mg (for cardiovascular diseases), osteoporosis (for Ca), will lead to the condition of weak bones and teeth (P), hypokalemia (K). The availability of these important micronutrients through the staple diet is a sustainable means to enhance global human capital. This is particularly important in the case of several countries in sub-Saharan Africa and South Asia where pearl millet is a major source of food, fodder and feed. To meet the micronutrient needs, developing nutrient-dense and agronomically desirable cultivars is an important objective in pearl millet breeding at ICRISAT and elsewhere. Pearl millet biofortification breeding program targets breeding pipelines which will have > 50% higher Fe and Zn content (+ 30 mg kg^−1^) over the existing hybrid parents and commercial varieties in Africa and India. To achieve such challenging breeding targets, exploitation of genetic variability present in the gene bank is a fast-track approach for the identification of mineral-dense accessions.

Core or mini core collection (10% of core collection or 1% of the entire collection)^16^ is a sound scientific strategy to systematically and cost-effectively screen the large germplasm collection conserved in the genebank globally. ICRISAT genebank conserves over 24000 accessions of pearl millet and its wild relatives (http://genebank.icrisat.org/ accessed on 5 Jan 2020). A core collection consisting of 2094 accessions that representing a diversity of the entire collection was developed by Upadhyaya et al.^15^. This study involved a part (504 accessions) of the core collection that was evaluated in two seasons at the ICRISAT Centre Patancheru, India for grain nutrients and two agronomic traits. Estimation of nutrients in the grains may be influenced by their availability in the soil in experimental fields. The estimated Fe and Zn content in the upper 30 cm soil in the experimental fields were above the critical so as not to limit crop growth^17^. Our results indicated that there was not any Fe and Zn deficiency in the soil and thus no impact on proper phenotyping. Also, balanced nutrition is needed to be emphasized to measure full genetic potential by applying respective micronutrients for creating uniform selection environments. A similar recommendation was reported for pearl millet biofortified hybrids^18^.

ANOVA of the 212 and 39 core collection accessions separately, indicated that the mean square attributable to genotypes was highly significant indicating adequate variability in the accessions of a core collection for micronutrients and agronomic traits (Tables 1 and 2). The mean squares attributable to the environment were also significant for all the micronutrients except Mn and TGW for 39 accessions (Tables 1 and 2) indicating that the two seasons that we used to evaluate the core collection accessions were different and adequate to differentiate the genotypes. The role of G × E interactions was important but much less than that of genotypes as the magnitude of the sum of squares due to genotypes was much greater than the G × E interaction (Tables 1 and 2).

High estimates of heritability were found for most of the micronutrients including Fe and Zn contents. The lower G × E interactions and high heritability for micronutrients suggested the possibility of a good response to selection. Similar high heritability and low G × E interaction for these traits have been reported in pearl millet^19–22^. Further, the relative performance of accessions did not markedly vary from one season to another which further confirmed that the G × E influences are not affecting ranking and selection of the accessions for micronutrients^23^.

The present study revealed the several-folds variability for most of the essential nutrients as well as agronomic traits such as TGW and days to 50% flowering (Table 3). Simultaneous improvement of different micronutrients and agronomic traits is possible when they are not negatively correlated to each other and with agronomic traits. Our study revealed that the Fe was positively correlated with Zn in both the seasons (r = 0.43–0.51, *p* < 0.01) and in the pooled (r = 0.46, *p* < 0.01; Table 6) among highly selected core collection 39 accessions. A similar positive and highly significant correlation between Fe and Zn observed among 212 accessions in both the seasons (r = 0.57–0.69, *p* < 0.01) and in the pooled (r = 0.64, *p* < 0.01), displaying that simultaneous selection for high-Fe and Zn densities could be very successful in pearl millet. Such a positive direction correlation between Fe and Zn has been reported in several pearl millet studies^14,19,21,24–30^. This tight linkage among Fe and Zn densities could be an indication of some physiological functions responsible for Fe and Zn uptake and translocation are governed by similar genes or do not have an antagonistic effect. For instance, QTLs identified for Fe and Zn contents are co-localized in pearl millet, thus hypothesizing a common transporting pathway^31^. The biofortification initiative led by HarvestPlus (a CGIAR program) recommended pearl millet biofortification breeding target is for Fe while Zn can be improved as associated trait^32^. The Zn content was positively correlated with Mo and S indicated that these nutrients could be combined in biofortified lines. Similar reports on the increase in Zn leading to an increase in the S in wheat grains^33,34^. The witness of highly significant and positive correlation of Mg with Na, P, K, S, Cu, and Mo imply Mg can be improved with these nutrients and Mg could be playing an important symbiotic role in assimilating these nutrients to grains). Bashir et al.^25^ reported a positive correlation between Mg with P and a non-significant correlation with Na in pearl millet, a study in rice reported a positive correlation between Mg with P and Cu^35^. Given the observed positive association of Ca with Fe and Mn, this will consistently increase these nutrient densities.

Flowering always had a positive correlation with all the nutrients, which shows the late-flowering accessions had a longer time to accumulate nutrients than early flowering accessions. This could be of an artifact that influence of reduced grain weight in late-flowering accessions. Thus, higher nutrient content is observed in small shrunken grains. Earlier studies in pearl millet reported a positive correlation between Fe and Zn with TGW^20,24^. In another study non-significant negative and positive correlation was observed between Fe and Zn with TGW^26^, while Bashir et al.^25^ reported none of the micronutrients showed significant correlation with TGW. In sorghum^36,37^ and wheat^38^ reported Fe and Zn had a negative correlation with TGW. These negative linkages can be broken in breeding populations using selective crosses involving contrasting genotypes and selection in larger segregating populations.

Ten top accessions of core collection were identified based on per se performance for each nutrient. Some of the accessions were promising for more than one nutrient. Considering the importance of Ni in nitrogen metabolism and fixation in crops, its adequacy critical for germination and crop growth^39^. However, an elevated level of Ni causes stunted growth, chlorosis, nutrient imbalance in crops, and health burdens to humans, thus lower level is desirable. The top 15 accessions with multiple nutrients (Table 5) were identified. These 15 accessions originated from five countries in Africa (Ghana, Burkina Faso, Niger, Zimbabwe) and Asia (India) indicating their geographical diversity. The clustering of 39 accessions (comes from 9 countries), indicating that these 15 accessions occurred in four of the five clusters. The seven accessions from India were clustered into two clusters; cluster 1 had four accessions from India and one from Ghana whereas cluster 2 had three accessions from India and one from Niger (Fig. 1b). These 15 multiple nutrients accession including six accessions with higher levels for eight or more nutrients, also showed wide variability for days to 50% flowering (42–64 days) and TGW (7–16 g) (Table 5). These germplasm resources can be exploited to improve the multiple nutrients in different maturity and seed size that are missing in elite pearl millet lines and hybrid parents. Using the core/mini core approach multiple nutrient germplasm accessions have been identified in peanut^40^, sorghum^41^, finger millet^42^, foxtail millet^43^, and kodo millet^44^, These core collection accessions offer diverse sources for traits introgression and wider scope for selection for the improvement of target micronutrients in breeding populations. Recently, the baseline for Fe and Zn has been established in the India national pearl millet cultivar release policy based on micronutrient levels in commercial cultivars. This is mandatory for both summer and rainy season cultivars. It is very important to note that all 39 accessions (of which, 13 were from India) were found to exceed the Indian pearl millet cultivars baseline of 42 mg kg^−1^ Fe and 32 mg kg^−1^ Zn. While, 17 accessions including eight accessions from India, exceeded the biofortification breeding Fe target (72 mg kg^−1^). Previous studies in pearl millet^14,24^ reported that high-Fe/Zn sources are largely or entirely from *iniadi* germplasm (early-maturing, larger grain-size collections from Togo regions) and no reports on the availability of Indian origin germplasm for higher micronutrients than *iniadi*. The present study revealed that there are 7 accessions from India that had 72–88 mg kg^−1^ Fe content and 42–62 mg kg^−1^ Zn content. This opens new opportunities for utilization in the initial crossing program for developing parental pipelines to meet the national minimum standards for these micronutrients^45^. Application genomic tools would help assess the genetic constitution and identify genetically dissimilar accessions among these multiple nutrient accessions. Their use in the breeding programs will help in the development of and dissemination of high Fe and Zn containing genotypes within high yielding breeding pipelines to address both food and nutritional insecurity in a target millet region. A limited quantity of seed of these lines is available from the ICRISAT Genebank under Standard Material Transfer Agreement.

## Materials and methods

### Plant materials and field evaluation

A set of 504 pearl millet accessions, that were part of the 2094 accessions of the core collection, originating from more than 25 countries representing major pearl millet growing regions of the world^15^ were used in this study (Supplementary Table S1). While developing the pearl millet core collection, duplicates were excluded and only distinct accessions were included^15^. These accessions were grouped into four groups based on similar phenology using days to 50% flowering from the Genebank database. Thus group 1 consisted of accessions that flowered in 40–60 days, group 2 in 61–70 days, group 3 in 71–80 days, and group 4 in 81–120 days. Quantity of seed received from gene bank was limited, thus, trials were evaluated in two sets following an augmented design^46^ in the 2009 summer season (February–June) with two controls, ICMB 88004 and ICTP 8203 at every 10th accessions at the ICRISAT, Patancheru, India (17.53°N latitude, 78.27°E longitude, and 545msl), in the alfisols. ICMB 88004 is a potential breeding line derived directly from germplasm and ICTP 8203 a popular variety derived from landraces^47,48^. Each accession was planted in one row of 4 m in length with the row-to-row spacing of 60 cm. The distance between plants within a row was maintained at 10 cm by thinning at 10–12 days after planting. A basal dose of diammonium phosphate at 100 kg ha^−1^ (i.e., 18 kg N and 46 kg P) was applied before planting with 100 kg ha^−1^ of urea (i.e., 46 kg N) side-dressed 15 days after planting. The field was manually weeded just after thinning and irrigated at every 10 days interval to protect from any moisture stress. At the time of panicle emergence, the main panicles of 6–8 plants in each plot were covered with parchment paper bags for selfing. All the selfed panicles were harvested at/or after physiological maturity and sundried for 10–15 days before threshing. Due to very late flowering (> 75 days), we could not harvest the grains from some accessions as the field was maintained till 110 days. Few accessions also had a poor self-seed set (< 10%). Seventy-six accessions that matured late and had poor seed set were not included and selfed seed from 428 accessions was used for grain micronutrient analysis as described below.

### Selection and re-evaluation in two seasons

Based on agronomic performance and high grain Fe content (> 70 mg kg^−1^), 212 accessions (Supplementary Table S2) out of 428 from 18 countries were selected for further field evaluations. These 428 accessions had 71–185 mg kg^−1^ Fe and 48–116 mg kg^−1^ Zn with days to 50% flowering ranging between 44 and 66 days in the 2009 summer season. The selected 212 accessions and two controls (ICTP 8203 and ICMB 88004) were planted in a randomized complete block design (RCBD) using 2 replications in the 2011 summer and rainy seasons at ICRISAT, Patancheru. Of the 212 accessions, a set of 39 accessions seeds (Supplementary Table S3) having high-Fe, 1000-grain weight (TGW) were analyzed for all-grain nutrients using ICP-OES at Waite Analytical laboratory, Adelaide University, Australia. The alfisol fields at the ICRISAT are precision fields with a gentle slope of 0.5%. The planting, plant spacing, and crop-management practices were similar as described above except the row to row spacing of 75 cm in the 2011 rainy season. Days to 50% flowering were recorded on a plot basis when at least 50% of plants in an accession had full exsertion of stigmas. Considering the high cost of self-seed production and good reliability of open-pollinated grain samples in pearl millet for estimation of nutrients^49^, about 8–10 random open-pollinated main panicles from each plot were harvested in a cloth bag at after physiological maturity. These were hand threshed and approximately 20 g of grains were collected from each plot for determining 1000-grain weight (TGW) and for use in grain micronutrient analysis (described below). For estimating TGW a random sample of 200 grains was used from each plot, weighed, and multiplied with a factor of five.

### Micronutrient analysis

Selfed-seed samples from 428 accessions in the 2009 rainy season were analyzed for grain micronutrients using Inductively Coupled Plasma Optical Emission Spectrometer (ICP-OES) following the method described by Wheal et al.^50^ at Waite Analytical laboratory, Adelaide University, Australia. Briefly, grain samples were oven-dried overnight at 85 °C prior to digestion, grounded enough to pass through a 1 mm stainless steel sieve using Christie and Norris hammer mill, and stored in screw-top polycarbonate vials. Grain samples were digested with di-acid (nitric and perchloric acid) mixture. After digestion, the volume of the digest was made to 25 mL using distilled water, and the content was agitated for 1 min by a vortex mixer. These digests were used for determination using ICP-OES. All 212 replicated accessions samples from the 2011 summer and rainy seasons were analyzed using X-ray Fluorescence (XRF) Spectrophotometry at ICRISAT, Patancheru, India. Destructive (wet lab) micronutrient analytical cost is very high, adding additional cost burdens to the breeding program when dealing with larger and replicated germplasm samples. Therefore, the present study explored the potential use of X-ray Fluorescence (XRF) Spectrophotometry for Fe and Zn content. XRF analysis, a scanning based method^51^ was used to estimate Fe and Zn. Briefly, XRF uses aluminium sample cups of 30 mm diameter, 36 mm depth and > 20 g of grain weight capacity, combined with polypropylene inner cups sealed at one end with 4 μm Poly-4 XRF sample film. Cups in a batch of 10 were filled with 8–12 g of grain and were shaken to distribute grain evenly within the cup, which was then loaded in the XRF instrument holder. The XRF method is highly reliable in pearl millet and the readings were positively and significantly correlated (r ≥ 0.90 for Fe and Zn) with the ICP method^51, 52^. Experiment field representative soil samples collected from the top 30 cm layer were analyzed for extractable Fe and Zn content using atomic absorption spectroscopy (AAS) as described by Lindsay and Norvell^53^.

### Statistical analyses

Analysis of variance (ANOVA) of all the trials was performed for both individual environments and pooled data using the PROC GLM procedure in SAS 14.1 software^54^. Genotypes (accessions) were considered as random and environment (season) as fixed. Variance attributable to genotypes (σ^2^g) in individual seasons and pooled over two seasons were estimated for all traits. In the pooled analysis, σ^2^ge) variance components were estimated for all traits. The homogeneity of error variance for different seasons was tested by Bartlett's test^55^, which is sensitive to departures from normality^56^. Broad-sense heritability (H^2^b) was estimated across environments using the following formula

$${\text{H}}^{2} {\text{b}} = {{\upsigma ^{2} {\text{g}}} \mathord{\left/ {\vphantom {{\upsigma ^{2} {\text{g}}} {\left[ {\upsigma ^{2} {\text{g + }}\left. {{{\left( {\upsigma ^{2} {\text{ge}}} \right)} \mathord{\left/ {\vphantom {{\left( {\upsigma ^{2} {\text{ge}}} \right)} {\text{e}}}} \right. \kern-\nulldelimiterspace} {\text{e}}}} \right) + \left( {\upsigma ^{2} {\text{error}}/{\text{re}}} \right)} \right]}}} \right. \kern-\nulldelimiterspace} {\left[ {\upsigma ^{2} {\text{g + }}\left. {{{\left( {\upsigma ^{2} {\text{ge}}} \right)} \mathord{\left/ {\vphantom {{\left( {\upsigma ^{2} {\text{ge}}} \right)} {\text{e}}}} \right. \kern-\nulldelimiterspace} {\text{e}}}} \right) + \left( {\upsigma ^{2} {\text{error}}/{\text{re}}} \right)} \right]}},$$

where σ^2^g is the genotypic variance, σ^2^ge is the genotype × environment interaction variance, and σ^2^error is the residual variance, r is the number of replications and e is the number of environments. The cluster analysis was carried out using R v3.5.1^57^ and the clustering of genotypes was performed using Ward’s^58^ methods. The significance of differences between cluster mean values were tested following the Newman–Keuls procedure^59,60^. Phenotypic correlations were estimated among all the traits in each environment and pooled data and between XRF and ICP for 39 accessions and tested for significance^56^. Promising core collection accessions for Fe, Zn, and other micronutrients were identified based on the per se performance.

## Supplementary information

Supplementary Information.
