# Publisher Correction: Exploring the genetic variability and diversity of pearl millet core collection germplasm for grain nutritional traits improvement

**DOI:** 10.1038/s41598-021-89008-7

**Published:** 2021-05-03

**Authors:** Mahalingam Govindaraj, Kedar N. Rai, Anand Kanatti, Hari D. Upadhyaya, Harshad Shivade, Aluri S. Rao

**Affiliations:** 1 International Crops Research Institute for the Semi-Arid Tropics (ICRISAT), Patancheru, 502 324 Telangana India; 2 University of Georgia, Athens, GA 30605 USA

Correction to: *Scientific Reports* 10.1038/s41598-020-77818-0, published online 03 December 2020

This Article contains errors in Table 5, where the entries in the rows “Minimum value” and “Maximum value” are not aligned correctly with their corresponding columns. The correct Table 5 appears below as Table 1.

**Table 1:** Core collection accessions with high values of multiple nutritional traits s based on evaluations in the 2011 summer and 2011 rainy seasons, Patancheru, India. Values in bold indicate higher levels of multiple nutrients except for Ni and Na where the lowest is desirable.

Accession	Country	Cluster no. Fig 1b	Fe	Zn	Mn	Cu	Mo	Ni	Ca	Mg	Na	K	P	S	50% flower	1000-grain weight
			mg kg^−1^												(d)	(g)
IP 3329	India	1	**78**	**62**	**14**	**7.3**	**1.9**	1.4	155	**1438**	14	**4800**	**4400**	**1533**	60	7
IP 3749	India	2	**78**	**56**	**17**	**5.9**	1.2	**0.8**	**204**	**1523**	13	4367	**4200**	**1577**	58	9
IP 4454	India	2	**91**	**57**	**16**	3.9	0.7	1.3	**212**	1327	18	3867	3300	1195	56	8
IP 5316	Niger	2	**82**	51	**17**	**6.1**	1.3	1.1	**235**	1280	18	**4400**	3650	**1400**	57	11
IP 7208	India	1	**93**	47	**16**	**5.9**	1	1.9	**180**	**1377**	16	**4433**	3700	1303	53	7
IP 7536	India	1	**82**	**57**	10	**7.2**	**1.5**	**0.7**	119	1317	13	**4900**	**4033**	1237	58	7
IP 9351	Ghana	4	71	**61**	**17**	**7.5**	**1.8**	1.1	**229**	**1837**	22	**4633**	**4500**	**1523**	46	14
IP 9407	Ghana	4	64	**59**	**16**	**5.6**	**2.2**	1.3	**179**	**1613**	14	**4400**	**4025**	**1638**	45	13
IP 9496	Ghana	4	**76**	**65**	**14**	5.5	**2.2**	1.4	**208**	1333	16	**4825**	3650	1353	42	15
IP 9572	Ghana	1	**72**	**61**	**12**	5.4	**1.8**	**0.9**	105	**1390**	12	**4967**	**4033**	**1455**	64	10
IP 11316	Burkina Faso	5	70	**58**	12	5.1	**1.5**	1.4	85	**1420**	**11**	3375	3400	**1530**	56	16
IP 11784	India	2	52	46	**16**	**6.1**	**1.3**	1.5	**164**	**1740**	20	**5133**	**4733**	**1703**	51	10
IP 12507	India	1	**76**	42	12	**6.7**	**1.5**	1.2	128	**1405**	15	**4775**	**4100**	**1583**	51	10
IP 12939	Ghana	4	**74**	**56**	11	**6.6**	**1.5**	1.2	**161**	**1453**	**10**	3767	**3800**	**1480**	43	13
IP 14148	Zimbabwe	5	69	55	12	5.3	1.2	**0.9**	**205**	1307	13	**4667**	**4100**	1298	59	10
Minimum value			52	42	10	4	1	1	85	1280	10	3375	3300	1195	42	7
Maximum value			93	65	17	7	2	2	235	1837	22	5133	4733	1703	64	16

