# Supplementary material for: Exploring the genetic variability and diversity of pearl millet core collection germplasm for grain nutritional traits improvement
Source: Sci Rep. 2020 Dec 3;10:21177. doi: 10.1038/s41598-020-77818-0 (PMC7713302; doi:10.1038/s41598-020-77818-0)
Supplement: Supplementary file 1 — Supplementary Information. [file 41598_2020_77818_MOESM1_ESM.pdf]

Exploring the genetic variability and diversity of pearl millet core collection germplasm for grain micronutrients improvement

Mahalingam Govindaraj[1*], Kedar N Rai[1], Anand Kanatti[1], Hari D Upadhyaya[1, 2], Harshad Shivade[1] and Aluri S Rao[1]

[1] International Crops Research Institute for the Semi-Arid Tropics (ICRISAT), Patancheru 502 324, Telangana, India.

[2]Center of Excellence for Advanced Materials Research, King Abdulaziz University Jeddah 21589, Saudi Arabia.

*Corresponding author (m.govindaraj@cgiar.org)

Supplimental Table S1. Pearl millet core collection accessions (504) originated from different countries obtained from Genebank, ICRISAT, Patancheru

S. No.	Accession identifier	Country of origin	Part of Core	Bloom (days) (Mean of two sets)
1	IP 3689	India	Yes	46
2	IP 3926	India	Yes	48
3	IP 3940	India	Yes	49
4	IP 3999	India	Yes	47
5	IP 4039	India	Yes	49
6	IP 4066	India	Yes	48
7	IP 4150	India	Yes	45
8	IP 4169	India	Yes	50
9	IP 4197	India	Yes	50
10	IP 4291	India	Yes	48
11	IP 4299	India	Yes	49
12	IP 4979	Nigeria	Yes	51
13	IP 6125	Cameroon	Yes	59
14	IP 6146	Cameroon	Yes	61
15	IP 6179	Cameroon	Yes	61
16	IP 6202	Cameroon	Yes	*
17	IP 6212	Cameroon	Yes	*
18	IP 6396	Mali	Yes	*
19	IP 6554	Mali	Yes	62
20	IP 6584	Malawi	Yes	56
21	IP 6604	Malawi	Yes	56
22	IP 6745	Malawi	Yes	55
23	IP 6769	Malawi	Yes	61
24	IP 6869	Kenya	Yes	61
25	IP 6882	Kenya	Yes	58
26	IP 7633	India	Yes	51
27	IP 9407	Ghana	Yes	49
28	IP 9416	Ghana	Yes	48
29	IP 9426	Ghana	Yes	50
30	IP 9840	Sudan	Yes	48
31	IP 10076	Mali	Yes	58
32	IP 10186	Mali	Yes	56
33	IP 10202	Mali	Yes	58
34	IP 10230	Mali	Yes	52
35	IP 10394	India	Yes	50
36	IP 10587	Mali	Yes	62
37	IP 10811	Sudan	Yes	56
38	IP 10820	Sudan	Yes	58
39	IP 10839	Sudan	Yes	58
40	IP 12570	India	Yes	45
41	IP 12650	India	Yes	47
42	IP 12901	Cameroon	Yes	59
43	IP 12925	Ghana	Yes	50
44	IP 12939	Ghana	Yes	46
45	IP 13150	Niger	Yes	60
46	IP 13557	India	Yes	51

Supplimental Table S1. Pearl millet core collection accessions (504) originated from different countries obtained from Genebank, ICRISAT, Patancheru

S. No.	Accession identifier	Country of origin	Part of Core	Bloom (days) (Mean of two sets)
47	IP 14926	Uganda	Yes	55
48	IP 14940	India	Yes	58
49	IP 14943	India	Yes	52
50	IP 14991	India	Yes	57
51	IP 15002	India	Yes	58
52	IP 15010	India	Yes	47
53	IP 15413	India	Yes	50
54	IP 15553	Burkina Faso	Yes	60
55	IP 17199	Burkina Faso	Yes	58
56	IP 17878	India	Yes	56
57	IP 18062	Pakistan	Yes	46
58	IP 18079	Pakistan	Yes	52
59	IP 18090	Pakistan	Yes	56
60	IP 18116	Pakistan	Yes	54
61	IP 18150	Pakistan	Yes	49
62	IP 18157	Mali	Yes	59
63	IP 18179	Mali	Yes	53
64	IP 18281	India	Yes	55
65	IP 18421	Namibia	Yes	60
66	IP 18535	Namibia	Yes	60
67	IP 18625	Namibia	Yes	57
68	IP 18657	Namibia	Yes	60
69	IP 18679	Namibia	Yes	57
70	IP 18780	Namibia	Yes	59
71	IP 18854	Namibia	Yes	60
72	IP 19040	Namibia	Yes	59
73	IP 19067	Namibia	Yes	58
74	IP 19072	Namibia	Yes	57
75	IP 19078	Namibia	Yes	60
76	IP 19141	Namibia	Yes	55
77	IP 19160	Namibia	Yes	58
78	IP 19175	Namibia	Yes	60
79	IP 19246	Namibia	Yes	59
80	IP 19263	Namibia	Yes	59
81	IP 19405	Chad	Yes	60
82	IP 19408	Chad	Yes	*
83	IP 3106	India	Yes	56
84	IP 3122	India	Yes	60
85	IP 3125	India	Yes	49
86	IP 3138	India	Yes	48
87	IP 3150	India	Yes	49
88	IP 3171	India	Yes	49
89	IP 3183	India	Yes	46
90	IP 3284	India	Yes	54
91	IP 3329	India	Yes	54
92	IP 3432	India	Yes	49

Supplimental Table S1. Pearl millet core collection accessions (504) originated from different countries obtained from Genebank, ICRISAT, Patancheru

S. No.	Accession identifier	Country of origin	Part of Core	Bloom (days) (Mean of two sets)
93	IP 3799	India	Yes	50
94	IP 3811	India	Yes	51
95	IP 3865	India	Yes	54
96	IP 3890	India	Yes	52
97	IP 3963	India	Yes	47
98	IP 4120	India	Yes	53
99	IP 4454	India	Yes	58
100	IP 4466	India	Yes	54
101	IP 4634	India	Yes	53
102	IP 4759	India	Yes	63
103	IP 4828	India	Yes	56
104	IP 4952	Uganda	Yes	59
105	IP 5389	Niger	Yes	64
106	IP 5900	Senegal	Yes	61
107	IP 6013	Senegal	Yes	61
108	IP 6060	Central African Republic	Yes	*
109	IP 6148	Cameroon	Yes	*
110	IP 6310	Mali	Yes	59
111	IP 6415	Mali	Yes	57
112	IP 6417	Mali	Yes	58
113	IP 6451	Mali	Yes	62
114	IP 6465	Mali	Yes	63
115	IP 6482	Mali	Yes	61
116	IP 6530	Mali	Yes	56
117	IP 6545	Mali	Yes	*
118	IP 6567	Mali	Yes	64
119	IP 6639	Malawi	Yes	61
120	IP 6682	Malawi	Yes	61
121	IP 6897	India	Yes	55
122	IP 7020	India	Yes	60
123	IP 7095	India	Yes	57
124	IP 7108	India	Yes	60
125	IP 7536	India	Yes	58
126	IP 7660	India	Yes	57
127	IP 7734	India	Yes	55
128	IP 7780	India	Yes	62
129	IP 7838	India	Yes	52
130	IP 8074	India	Yes	58
131	IP 8208	India	Yes	57
132	IP 8229	India	Yes	57
133	IP 8237	India	Yes	60
134	IP 8339	India	Yes	58
135	IP 8344	India	Yes	57
136	IP 8350	India	Yes	57
137	IP 8426	Nigeria	Yes	58
138	IP 9077	India	Yes	58

Supplimental Table S1. Pearl millet core collection accessions (504) originated from different countries obtained from Genebank, ICRISAT, Patancheru

S. No.	Accession identifier	Country of origin	Part of Core	Bloom (days) (Mean of two sets)
139	IP 9242	Mali	Yes	63
140	IP 9286	Togo	Yes	62
141	IP 9301	Togo	Yes	52
142	IP 9351	Ghana	Yes	50
143	IP 9496	Ghana	Yes	46
144	IP 9572	Ghana	Yes	55
145	IP 9969	Zambia	Yes	60
146	IP 9971	Zambia	Yes	63
147	IP 9981	Zambia	Yes	61
148	IP 10085	Mali	Yes	57
149	IP 10140	Mali	Yes	*
150	IP 10273	Nigeria	Yes	64
151	IP 10369	Nigeria	Yes	61
152	IP 10401	India	Yes	*
153	IP 10410	Togo	Yes	*
154	IP 10471	Zimbabwe	Yes	63
155	IP 10539	Senegal	Yes	63
156	IP 10543	Mali	Yes	65
157	IP 10643	Mali	Yes	65
158	IP 10652	Mali	Yes	62
159	IP 10694	Mali	Yes	63
160	IP 10761	Sudan	Yes	63
161	IP 10885	Sudan	Yes	63
162	IP 11049	India	Yes	58
163	IP 11318	Burkina Faso	Yes	57
164	IP 11358	Burkina Faso	Yes	*
165	IP 11384	Burkina Faso	Yes	62
166	IP 11389	Burkina Faso	Yes	59
167	IP 11391	Burkina Faso	Yes	57
168	IP 11503	Burkina Faso	Yes	56
169	IP 11505	Burkina Faso	Yes	56
170	IP 11577	Burkina Faso	Yes	56
171	IP 11584	Burkina Faso	Yes	60
172	IP 11680	Sudan	Yes	57
173	IP 11784	India	Yes	52
174	IP 11875	India	Yes	49
175	IP 11893	India	Yes	58
176	IP 11961	Nigeria	Yes	61
177	IP 11975	Nigeria	Yes	61
178	IP 12111	Nigeria	Yes	55
179	IP 12116	Nigeria	Yes	58
180	IP 12138	Nigeria	Yes	*
181	IP 12181	Nigeria	Yes	63
182	IP 12248	Nigeria	Yes	63
183	IP 12282	Nigeria	Yes	60
184	IP 12310	Nigeria	Yes	58

Supplimental Table S1. Pearl millet core collection accessions (504) originated from different countries obtained from Genebank, ICRISAT, Patancheru

S. No.	Accession identifier	Country of origin	Part of Core	Bloom (days) (Mean of two sets)
185	IP 12532	India	Yes	54
186	IP 12568	India	Yes	59
187	IP 12591	India	Yes	59
188	IP 12700	India	Yes	55
189	IP 12768	India	Yes	50
190	IP 12805	India	Yes	52
191	IP 12839	Botswana	Yes	64
192	IP 12967	Malawi	Yes	51
193	IP 13137	Niger	Yes	56
194	IP 13154	Niger	Yes	*
195	IP 13180	Nigeria	Yes	63
196	IP 13290	Senegal	Yes	64
197	IP 13384	Uganda	Yes	53
198	IP 13459	India	Yes	57
199	IP 13482	India	Yes	58
200	IP 13565	India	Yes	58
201	IP 13645	India	Yes	56
202	IP 13900	Burkina Faso	Yes	46
203	IP 14294	Cameroon	Yes	*
204	IP 14311	Cameroon	Yes	*
205	IP 14317	Cameroon	Yes	*
206	IP 14362	Cameroon	Yes	*
207	IP 14418	Cameroon	Yes	*
208	IP 14426	Cameroon	Yes	*
209	IP 14497	Cameroon	Yes	*
210	IP 14624	Cameroon	Yes	*
211	IP 14644	Cameroon	Yes	*
212	IP 14787	Cameroon	Yes	*
213	IP 14849	Cameroon	Yes	*
214	IP 14923	Uganda	Yes	*
215	IP 14942	India	Yes	53
216	IP 15033	India	Yes	50
217	IP 15256	India	Yes	50
218	IP 15273	India	Yes	57
219	IP 15297	India	Yes	46
220	IP 15304	India	Yes	55
221	IP 15355	India	Yes	52
222	IP 15390	India	Yes	55
223	IP 15402	India	Yes	50
224	IP 15423	India	Yes	53
225	IP 15438	India	Yes	55
226	IP 15498	India	Yes	50
227	IP 15556	Burkina Faso	Yes	*
228	IP 15598	Burkina Faso	Yes	56
229	IP 15610	Burkina Faso	Yes	*
230	IP 15917	Togo	Yes	47

Supplimental Table S1. Pearl millet core collection accessions (504) originated from different countries obtained from Genebank, ICRISAT, Patancheru

S. No.	Accession identifier	Country of origin	Part of Core	Bloom (days) (Mean of two sets)
231	IP 15953	India	Yes	59
232	IP 16009	India	Yes	49
233	IP 16096	India	Yes	48
234	IP 16197	India	Yes	46
235	IP 16212	India	Yes	52
236	IP 17217	Burkina Faso	Yes	56
237	IP 17314	Burkina Faso	Yes	60
238	IP 17350	Burkina Faso	Yes	67
239	IP 17360	Burkina Faso	Yes	65
240	IP 17462	Central African Republic	Yes	*
241	IP 17493	Togo	Yes	*
242	IP 17566	Togo	Yes	44
243	IP 17632	Togo	Yes	47
244	IP 17707	Togo	Yes	47
245	IP 17978	India	Yes	46
246	IP 17979	India	No	49
247	IP 18168	Mali	Yes	55
248	IP 18246	India	Yes	60
249	IP 18389	Namibia	Yes	63
250	IP 18500	Namibia	Yes	65
251	IP 18621	Namibia	Yes	*
252	IP 18702	Namibia	Yes	63
253	IP 18742	Namibia	Yes	62
254	IP 18786	Namibia	Yes	*
255	IP 18797	Namibia	Yes	65
256	IP 18800	Namibia	Yes	62
257	IP 18910	Namibia	Yes	68
258	IP 18988	Namibia	Yes	65
259	IP 19190	Namibia	Yes	62
260	IP 19215	Namibia	Yes	64
261	IP 19229	Namibia	Yes	65
262	IP 19243	Namibia	Yes	*
263	IP 19299	Namibia	Yes	63
264	IP 19305	Namibia	Yes	*
265	IP 19321	Namibia	Yes	63
266	IP 19406	Chad	Yes	62
267	IP 3133	India	Yes	49
268	IP 3163	India	Yes	48
269	IP 3296	India	Yes	50
270	IP 3382	India	Yes	60
271	IP 3389	India	Yes	50
272	IP 3529	India	Yes	60
273	IP 3557	India	Yes	60
274	IP 3626	India	Yes	56
275	IP 3646	India	Yes	56
276	IP 3749	India	Yes	56

Supplimental Table S1. Pearl millet core collection accessions (504) originated from different countries obtained from Genebank, ICRISAT, Patancheru

S. No.	Accession identifier	Country of origin	Part of Core	Bloom (days) (Mean of two sets)
277	IP 4331	India	Yes	62
278	IP 4499	India	Yes	58
279	IP 4542	India	Yes	58
280	IP 4695	India	Yes	58
281	IP 4747	India	Yes	59
282	IP 4749	India	Yes	62
283	IP 4779	India	Yes	58
284	IP 4962	Uganda	Yes	58
285	IP 4965	Uganda	Yes	56
286	IP 5045	Nigeria	Yes	58
287	IP 5272	Niger	Yes	58
288	IP 5275	Niger	Yes	57
289	IP 5316	Niger	Yes	57
290	IP 5438	Niger	Yes	60
291	IP 5441	Niger	Yes	58
292	IP 5477	Niger	Yes	60
293	IP 5816	Senegal	Yes	65
294	IP 5873	Senegal	Yes	60
295	IP 5940	Senegal	Yes	62
296	IP 6037	Central African Republic	Yes	*
297	IP 6460	Mali	Yes	60
298	IP 6461	Mali	Yes	60
299	IP 6510	Mali	Yes	*
300	IP 7112	India	Yes	58
301	IP 7118	India	Yes	59
302	IP 7208	India	Yes	57
303	IP 7470	Tanzania	Yes	*
304	IP 7487	Tanzania	Yes	*
305	IP 7762	India	Yes	53
306	IP 8086	India	Yes	56
307	IP 8130	India	Yes	58
308	IP 8198	Nigeria	Yes	59
309	IP 8276	India	Yes	56
310	IP 8409	Nigeria	Yes	58
311	IP 8472	Niger	Yes	65
312	IP 8562	India	Yes	60
313	IP 8564	India	Yes	62
314	IP 8593	India	Yes	50
315	IP 8609	India	Yes	50
316	IP 8867	Zambia	Yes	55
317	IP 8916	Senegal	Yes	57
318	IP 8972	Togo	Yes	50
319	IP 9140	India	Yes	60
320	IP 9149	India	Yes	59
321	IP 9595	Yemen	Yes	62
322	IP 9692	Nigeria	Yes	64

Supplimental Table S1. Pearl millet core collection accessions (504) originated from different countries obtained from Genebank, ICRISAT, Patancheru

S. No.	Accession identifier	Country of origin	Part of Core	Bloom (days) (Mean of two sets)
323	IP 9710	Nigeria	Yes	60
324	IP 9720	Nigeria	Yes	58
325	IP 10300	Nigeria	Yes	65
326	IP 10339	Nigeria	Yes	57
327	IP 10437	Benin	Yes	*
328	IP 10456	Zimbabwe	Yes	60
329	IP 10486	Zimbabwe	Yes	60
330	IP 10488	Zimbabwe	Yes	59
331	IP 10705	Mali	Yes	56
332	IP 11143	India	Yes	55
333	IP 11144	India	Yes	49
334	IP 11211	India	Yes	56
335	IP 11275	Burkina Faso	Yes	55
336	IP 11310	Burkina Faso	Yes	*
337	IP 11316	Burkina Faso	Yes	60
338	IP 11320	Burkina Faso	Yes	52
339	IP 11353	Burkina Faso	Yes	*
340	IP 11537	Burkina Faso	Yes	56
341	IP 11593	Burkina Faso	Yes	65
342	IP 11677	Sudan	Yes	56
343	IP 12020	Nigeria	Yes	*
344	IP 12331	Nigeria	Yes	60
345	IP 12338	Nigeria	Yes	63
346	IP 12395	South Africa	Yes	60
347	IP 12464	India	Yes	55
348	IP 12474	India	Yes	55
349	IP 12507	India	Yes	49
350	IP 12627	India	Yes	60
351	IP 12669	India	Yes	57
352	IP 12682	India	Yes	61
353	IP 12779	India	Yes	58
354	IP 13075	Niger	Yes	60
355	IP 13121	Niger	Yes	62
356	IP 13191	Nigeria	Yes	64
357	IP 13196	Nigeria	Yes	58
358	IP 13280	Senegal	Yes	62
359	IP 13370	Tanzania	Yes	*
360	IP 13833	Burkina Faso	Yes	50
361	IP 13875	Burkina Faso	Yes	*
362	IP 13878	Burkina Faso	Yes	64
363	IP 13907	Niger	Yes	56
364	IP 13964	Zimbabwe	Yes	58
365	IP 14026	Zimbabwe	Yes	58
366	IP 14038	Zimbabwe	Yes	60
367	IP 14148	Zimbabwe	Yes	55
368	IP 14210	Cameroon	Yes	64

Supplimental Table S1. Pearl millet core collection accessions (504) originated from different countries obtained from Genebank, ICRISAT, Patancheru

S. No.	Accession identifier	Country of origin	Part of Core	Bloom (days) (Mean of two sets)
369	IP 14217	Cameroon	Yes	*
370	IP 14778	Cameroon	Yes	64
371	IP 14918	Uganda	Yes	*
372	IP 15159	India	Yes	56
373	IP 15177	India	Yes	*
374	IP 15218	India	Yes	58
375	IP 15220	India	Yes	56
376	IP 15233	India	Yes	60
377	IP 15234	India	Yes	60
378	IP 15257	India	Yes	57
379	IP 15614	Burkina Faso	Yes	*
380	IP 15817	Tanzania	Yes	59
381	IP 15872	Tanzania	Yes	*
382	IP 15899	Tanzania	Yes	56
383	IP 16131	India	Yes	59
384	IP 16440	Zimbabwe	Yes	60
385	IP 16638	Zimbabwe	Yes	48
386	IP 17028	Zimbabwe	Yes	59
387	IP 17099	Zimbabwe	Yes	58
388	IP 17125	Zimbabwe	Yes	60
389	IP 17228	Burkina Faso	Yes	55
390	IP 17407	Central African Republic	Yes	*
391	IP 17435	Central African Republic	Yes	*
392	IP 17554	Togo	Yes	49
393	IP 17620	Togo	Yes	50
394	IP 17690	Togo	Yes	50
395	IP 17753	Togo	Yes	50
396	IP 17945	Nigeria	Yes	56
397	IP 18412	Namibia	Yes	58
398	IP 18722	Namibia	Yes	*
399	IP 19344	Namibia	Yes	60
400	IP 19361	Namibia	Yes	58
401	IP 19388	Namibia	Yes	*
402	IP 3098	India	Yes	*
403	IP 4974	Nigeria	Yes	57
404	IP 5031	Nigeria	Yes	59
405	IP 5056	Nigeria	Yes	58
406	IP 5070	Nigeria	Yes	56
407	IP 5121	Niger	Yes	57
408	IP 5131	Niger	Yes	60
409	IP 5153	Niger	Yes	*
410	IP 5207	Niger	Yes	55
411	IP 5221	Niger	Yes	55
412	IP 5222	Niger	Yes	57
413	IP 5308	Niger	Yes	63
414	IP 5396	Niger	Yes	*

Supplimental Table S1. Pearl millet core collection accessions (504) originated from different countries obtained from Genebank, ICRISAT, Patancheru

S. No.	Accession identifier	Country of origin	Part of Core	Bloom (days) (Mean of two sets)
415	IP 5416	Niger	Yes	60
416	IP 5419	Niger	Yes	65
417	IP 5447	Niger	Yes	60
418	IP 5452	Niger	Yes	64
419	IP 5455	Niger	Yes	60
420	IP 5597	Niger	Yes	*
421	IP 5603	Niger	Yes	68
422	IP 5666	Niger	Yes	56
423	IP 5695	Nigeria	Yes	*
424	IP 5713	Nigeria	Yes	59
425	IP 6585	Malawi	Yes	55
426	IP 6793	Malawi	Yes	57
427	IP 7364	Tanzania	Yes	*
428	IP 7377	Tanzania	Yes	*
429	IP 7392	Tanzania	Yes	*
430	IP 7440	Tanzania	Yes	*
431	IP 7468	Tanzania	Yes	*
432	IP 7886	India	Yes	*
433	IP 7915	Niger	Yes	*
434	IP 7935	India	Yes	65
435	IP 7952	India	Yes	*
436	IP 8000	Botswana	Yes	*
437	IP 8069	India	Yes	*
438	IP 8429	Nigeria	Yes	56
439	IP 8540	India	Yes	*
440	IP 8647	Sudan	Yes	*
441	IP 8672	Sudan	Yes	*
442	IP 8786	Botswana	Yes	58
443	IP 8818	Zimbabwe	Yes	64
444	IP 8863	Zambia	Yes	*
445	IP 9618	India	Yes	50
446	IP 9678	Nigeria	Yes	56
447	IP 10290	Nigeria	Yes	*
448	IP 10423	Benin	Yes	*
449	IP 10912	Sudan	Yes	*
450	IP 10945	Sudan	Yes	60
451	IP 10953	Kenya	Yes	51
452	IP 10964	Kenya	Yes	50
453	IP 11218	Zimbabwe	Yes	*
454	IP 11311	Burkina Faso	Yes	54
455	IP 11387	Burkina Faso	Yes	65
456	IP 11454	Burkina Faso	Yes	66
457	IP 11707	Sudan	Yes	65
458	IP 11763	South Africa	Yes	*
459	IP 11765	South Africa	Yes	*
460	IP 11841	India	Yes	*

Supplimental Table S1. Pearl millet core collection accessions (504) originated from different countries obtained from Genebank, ICRISAT, Patancheru

S. No.	Accession identifier	Country of origin	Part of Core	Bloom (days) (Mean of two sets)
461	IP 11930	Sierra Leone	Yes	*
462	IP 11937	Sierra Leone	Yes	*
463	IP 11947	Sierra Leone	Yes	*
464	IP 12848	Burkina Faso	Yes	62
465	IP 13608	India	Yes	57
466	IP 13840	Burkina Faso	Yes	*
467	IP 13885	Burkina Faso	Yes	*
468	IP 13892	Burkina Faso	Yes	*
469	IP 13971	Zimbabwe	Yes	*
470	IP 14028	Zimbabwe	Yes	58
471	IP 14100	Zimbabwe	Yes	60
472	IP 14160	Zimbabwe	Yes	64
473	IP 14172	Zimbabwe	Yes	65
474	IP 15092	India	Yes	*
475	IP 15372	India	Yes	57
476	IP 15681	Tanzania	Yes	*
477	IP 15710	Tanzania	Yes	*
478	IP 15829	Tanzania	Yes	*
479	IP 15857	Tanzania	Yes	*
480	IP 16289	Zimbabwe	Yes	*
481	IP 16304	Zimbabwe	Yes	59
482	IP 16380	Zimbabwe	Yes	*
483	IP 16402	Zimbabwe	Yes	59
484	IP 16420	Zimbabwe	Yes	60
485	IP 16422	Zimbabwe	Yes	58
486	IP 16426	Zimbabwe	Yes	63
487	IP 16449	Zimbabwe	Yes	59
488	IP 16569	Zimbabwe	Yes	58
489	IP 16580	Zimbabwe	Yes	60
490	IP 16613	Zimbabwe	Yes	60
491	IP 16617	Zimbabwe	Yes	62
492	IP 16690	Zimbabwe	Yes	*
493	IP 16770	Zimbabwe	Yes	59
494	IP 16791	Zimbabwe	Yes	65
495	IP 16820	Zimbabwe	Yes	60
496	IP 16911	Zimbabwe	Yes	66
497	IP 16937	Zimbabwe	Yes	61
498	IP 16968	Zimbabwe	Yes	57
499	IP 17006	Zimbabwe	Yes	*
500	IP 17064	Zimbabwe	Yes	58
501	IP 17080	Zimbabwe	Yes	*
502	IP 17144	Zimbabwe	Yes	56
503	IP 18308	India	Yes	*
504	IP 18318	India	Yes	*

* Very late bloom (>70 d) and data not recorded

Supplimental Table S2. Selected 212 accessions with country of origin and field performance over two crop season

S. No.	Accession identifier	Country of origin	Part of Mini Core	Mean of Summer 2011 and Rainy 2011			
				Bloom (days)	TSW (g/1000 grain)	Fe_XRF (mg kg^{-1})	Zn_XRF (mg kg^{-1})
1	IP 3106	India	NO	54	11	59	59
2	IP 3122	India	NO	56	8	45	37
3	IP 3138	India	NO	50	7	44	48
4	IP 3163	India	NO	51	7	50	48
5	IP 3183	India	NO	48	7	37	42
6	IP 3284	India	NO	56	8	48	43
7	IP 3296	India	NO	50	11	55	58
8	IP 3329	India	YES	60	7	72	61
9	IP 3432	India	YES	53	7	44	64
10	IP 3626	India	YES	52	9	70	58
11	IP 3749	India	NO	55	9	59	65
12	IP 3799	India	NO	53	12	64	61
13	IP 3865	India	NO	55	7	44	50
14	IP 3890	India	NO	51	7	44	48
15	IP 3999	India	NO	46	10	41	44
16	IP 4120	India	NO	54	10	47	43
17	IP 4197	India	NO	57	11	64	47
18	IP 4291	India	YES	46	12	46	43
19	IP 4454	India	NO	56	9	67	66
20	IP 4466	India	NO	57	9	39	43
21	IP 4499	India	NO	57	7	42	56
22	IP 4542	India	NO	50	7	51	57
23	IP 4695	India	NO	59	7	49	37
24	IP 4747	India	YES	61	9	75	66
25	IP 4759	India	NO	53	9	54	45
26	IP 4779	India	NO	52	7	62	45
27	IP 4828	India	NO	56	9	48	46
28	IP 4962	Uganda	NO	52	12	57	41
29	IP 4974	Nigeria	NO	57	8	48	46
30	IP 4979	Nigeria	YES	52	11	46	52
31	IP 5031	Nigeria	NO	63	7	48	42
32	IP 5045	Nigeria	NO	52	12	55	47
33	IP 5056	Nigeria	NO	59	9	59	47
34	IP 5070	Nigeria	NO	55	11	59	52
35	IP 5131	Niger	NO	59	10	51	47
36	IP 5222	Niger	NO	54	9	54	46
37	IP 5272	Niger	NO	56	8	50	43
38	IP 5316	Niger	NO	57	11	60	50
39	IP 5666	Niger	NO	52	10	49	41
40	IP 5695	Nigeria	NO	54	7	38	34
41	IP 6013	Senegal	NO	55	10	51	46

Supplimental Table S2. Selected 212 accessions with country of origin and field performance over two crop season

S. No.	Accession identifier	Country of origin	Part of Mini Core	Mean of Summer 2011 and Rainy 2011			
				Bloom (days)	TSW (g/1000 grain)	Fe_XRF (mg kg^{-1})	Zn_XRF (mg kg^{-1})
42	IP 6530	Mali	NO	50	12	52	41
43	IP 6584	Malawi	NO	51	8	50	63
44	IP 6639	Malawi	NO	53	11	53	50
45	IP 6682	Malawi	NO	62	8	35	47
46	IP 6745	Malawi	NO	50	12	58	52
47	IP 6793	Malawi	NO	52	7	52	50
48	IP 6869	Kenya	NO	59	9	57	68
49	IP 6882	Kenya	NO	60	7	47	50
50	IP 6897	India	NO	60	8	44	45
51	IP 7020	India	NO	52	9	50	47
52	IP 7108	India	NO	58	8	55	56
53	IP 7118	India	YES	62	9	64	51
54	IP 7208	India	NO	53	7	69	54
55	IP 7377	Tanzania	NO	60	8	44	40
56	IP 7440	Tanzania	NO	55	8	40	33
57	IP 7470	Tanzania	NO	60	9	56	49
58	IP 7536	India	NO	58	7	72	51
59	IP 7633	India	NO	44	11	52	48
60	IP 7660	India	NO	56	9	49	48
61	IP 7734	India	NO	53	9	67	59
62	IP 7762	India	NO	57	8	49	39
63	IP 7838	India	NO	52	9	83	47
64	IP 8069	India	NO	69	9	38	43
65	IP 8074	India	YES	49	8	41	38
66	IP 8208	India	NO	49	11	60	43
67	IP 8339	India	NO	53	10	46	43
68	IP 8344	India	NO	53	7	57	51
69	IP 8409	Nigeria	NO	59	8	35	41
70	IP 8426	Nigeria	NO	59	9	73	54
71	IP 8429	Nigeria	NO	58	8	61	56
72	IP 8562	India	YES	60	9	47	44
73	IP 8564	India	NO	59	9	54	54
74	IP 8593	India	NO	54	10	45	35
75	IP 8863	Zambia	YES	63	8	41	47
76	IP 8867	Zambia	NO	49	8	58	47
77	IP 8972	Togo	NO	46	11	90	48
78	IP 9140	India	NO	59	8	49	64
79	IP 9286	Togo	NO	66	12	76	48
80	IP 9301	Togo	NO	49	14	83	59
81	IP 9351	Ghana	NO	46	15	69	51
82	IP 9407	Ghana	NO	45	14	66	48

Supplimental Table S2. Selected 212 accessions with country of origin and field performance over two crop season

S. No.	Accession identifier	Country of origin	Part of Mini Core	Mean of Summer 2011 and Rainy 2011			
				Bloom (days)	TSW (g/1000 grain)	Fe_XRF (mg kg^{-1})	Zn_XRF (mg kg^{-1})
83	IP 9416	Ghana	NO	45	14	70	54
84	IP 9426	Ghana	NO	55	8	56	53
85	IP 9496	Ghana	NO	42	15	74	46
86	IP 9572	Ghana	NO	64	9	45	47
87	IP 9618	India	NO	51	6	48	42
88	IP 9678	Nigeria	NO	64	9	70	56
89	IP 9840	Sudan	NO	53	10	46	43
90	IP 9969	Zambia	NO	56	6	60	62
91	IP 9981	Zambia	NO	59	8	47	51
92	IP 10202	Mali	NO	62	9	48	44
93	IP 10290	Nigeria	NO	61	7	58	44
94	IP 10394	India	NO	51	14	75	47
95	IP 10471	Zimbabwe	NO	61	7	60	55
96	IP 10543	Mali	NO	60	10	41	37
97	IP 10694	Mali	NO	63	7	39	34
98	IP 10761	Sudan	YES	61	8	39	39
99	IP 10811	Sudan	NO	57	10	69	50
100	IP 10820	Sudan	NO	59	9	41	38
101	IP 10964	Kenya	NO	57	8	69	73
102	IP 11049	India	NO	53	6	42	47
103	IP 11316	Burkina Faso	NO	56	15	63	54
104	IP 11320	Burkina Faso	NO	47	15	45	43
105	IP 11353	Burkina Faso	NO	67	13	46	47
106	IP 11358	Burkina Faso	NO	60	7	46	33
107	IP 11391	Burkina Faso	NO	57	10	39	41
108	IP 11537	Burkina Faso	NO	53	9	46	38
109	IP 11584	Burkina Faso	NO	60	11	64	65
110	IP 11677	Sudan	NO	53	6	62	57
111	IP 11784	India	NO	51	10	65	51
112	IP 11875	India	NO	48	10	57	42
113	IP 11893	India	NO	59	10	67	61
114	IP 12020	Nigeria	NO	64	9	56	42
115	IP 12138	Nigeria	NO	67	7	57	51
116	IP 12181	Nigeria	NO	63	9	57	60
117	IP 12507	India	NO	50	10	65	44
118	IP 12627	India	NO	65	10	59	57
119	IP 12682	India	NO	58	10	68	50
120	IP 12768	India	NO	53	7	70	52
121	IP 12805	India	YES	51	8	48	45
122	IP 12901	Cameroon	NO	61	8	45	44
123	IP 12925	Ghana	NO	51	14	78	50

Supplimental Table S2. Selected 212 accessions with country of origin and field performance over two crop season

S. No.	Accession identifier	Country of origin	Part of Mini Core	Mean of Summer 2011 and Rainy 2011			
				Bloom (days)	TSW (g/1000 grain)	Fe_XRF (mg kg^{-1})	Zn_XRF (mg kg^{-1})
124	IP 12939	Ghana	NO	46	13	68	59
125	IP 12967	Malawi	NO	50	7	56	45
126	IP 13137	Niger	NO	54	12	38	34
127	IP 13154	Niger	NO	62	10	40	39
128	IP 13180	Nigeria	NO	56	11	56	46
129	IP 13191	Nigeria	NO	58	12	59	58
130	IP 13290	Senegal	NO	62	8	44	45
131	IP 13384	Uganda	NO	55	11	58	46
132	IP 13565	India	NO	59	6	56	65
133	IP 13900	Burkina Faso	NO	51	12	61	56
134	IP 13971	Zimbabwe	NO	60	11	46	47
135	IP 14026	Zimbabwe	NO	53	10	58	49
136	IP 14028	Zimbabwe	NO	56	8	37	45
137	IP 14148	Zimbabwe	NO	54	10	61	47
138	IP 14210	Cameroon	NO	53	12	59	53
139	IP 14362	Cameroon	NO	64	8	56	64
140	IP 14418	Cameroon	NO	63	7	42	51
141	IP 14778	Cameroon	NO	52	11	45	39
142	IP 14942	India	NO	48	5	41	49
143	IP 14991	India	NO	51	10	46	50
144	IP 15092	India	NO	56	9	70	74
145	IP 15159	India	NO	56	8	43	47
146	IP 15177	India	NO	53	11	36	54
147	IP 15218	India	NO	63	7	47	60
148	IP 15233	India	NO	58	7	45	54
149	IP 15234	India	NO	56	8	62	50
150	IP 15257	India	NO	61	7	47	57
151	IP 15273	India	YES	49	7	68	42
152	IP 15297	India	NO	47	7	53	48
153	IP 15304	India	NO	57	6	48	44
154	IP 15372	India	YES	50	7	43	36
155	IP 15402	India	NO	54	9	70	50
156	IP 15423	India	NO	55	11	46	40
157	IP 15498	India	NO	53	11	49	40
158	IP 15614	Burkina Faso	NO	59	6	63	56
159	IP 15817	Tanzania	NO	60	9	81	47
160	IP 15872	Tanzania	NO	63	6	38	36
161	IP 15899	Tanzania	NO	58	6	58	54
162	IP 15917	Togo	NO	45	13	85	48
163	IP 16197	India	NO	48	12	44	38
164	IP 16304	Zimbabwe	NO	61	7	36	45

Supplimental Table S2. Selected 212 accessions with country of origin and field performance over two crop season

S. No.	Accession identifier	Country of origin	Part of Mini Core	Mean of Summer 2011 and Rainy 2011			
				Bloom (days)	TSW (g/1000 grain)	Fe_XRF (mg kg^{-1})	Zn_XRF (mg kg^{-1})
165	IP 16380	Zimbabwe	NO	59	9	47	43
166	IP 16426	Zimbabwe	NO	62	7	44	44
167	IP 16449	Zimbabwe	NO	43	11	56	51
168	IP 16617	Zimbabwe	NO	57	10	58	53
169	IP 16770	Zimbabwe	NO	54	9	64	49
170	IP 16911	Zimbabwe	NO	58	9	56	53
171	IP 17028	Zimbabwe	NO	58	10	59	58
172	IP 17064	Zimbabwe	NO	58	10	64	61
173	IP 17125	Zimbabwe	NO	58	7	40	49
174	IP 17217	Burkina Faso	NO	60	9	66	65
175	IP 17554	Togo	NO	44	13	59	36
176	IP 17566	Togo	NO	41	14	66	49
177	IP 17620	Togo	NO	53	13	88	59
178	IP 17632	Togo	NO	41	14	57	39
179	IP 17690	Togo	NO	53	14	82	57
180	IP 17707	Togo	NO	50	12	76	54
181	IP 17878	India	NO	50	13	80	55
182	IP 17978	India	NO	47	12	64	55
183	IP 18246	India	NO	59	7	68	68
184	IP 18281	India	NO	58	11	56	48
185	IP 18389	Namibia	NO	55	13	58	51
186	IP 18421	Namibia	NO	58	10	54	51
187	IP 18500	Namibia	NO	60	10	45	41
188	IP 18621	Namibia	NO	63	8	44	43
189	IP 18657	Namibia	YES	51	6	45	47
190	IP 18679	Namibia	NO	60	8	43	45
191	IP 18702	Namibia	NO	58	9	39	38
192	IP 18722	Namibia	NO	60	8	52	50
193	IP 18742	Namibia	NO	48	10	55	46
194	IP 18780	Namibia	NO	61	8	45	43
195	IP 18786	Namibia	NO	65	8	57	53
196	IP 18797	Namibia	NO	61	8	57	54
197	IP 18910	Namibia	NO	55	10	66	52
198	IP 19040	Namibia	NO	66	8	44	44
199	IP 19067	Namibia	NO	61	9	42	40
200	IP 19072	Namibia	YES	58	7	43	37
201	IP 19078	Namibia	NO	57	7	34	30
202	IP 19141	Namibia	YES	51	9	40	43
203	IP 19175	Namibia	NO	56	12	51	51
204	IP 19190	Namibia	NO	56	14	64	49
205	IP 19215	Namibia	NO	60	8	56	52

Supplimental Table S2. Selected 212 accessions with country of origin and field performance over two crop season

S. No.	Accession identifier	Country of origin	Part of Mini Core	Mean of Summer 2011 and Rainy 2011			
				Bloom (days)	TSW (g/1000 grain)	Fe_XRF (mg kg^{-1})	Zn_XRF (mg kg^{-1})
206	IP 19229	Namibia	NO	67	8	48	47
207	IP 19263	Namibia	NO	58	11	41	39
208	IP 19299	Namibia	NO	66	9	47	40
209	IP 19305	Namibia	YES	68	8	46	43
210	IP 19344	Namibia	NO	62	9	37	37
211	IP 19388	Namibia	NO	70	8	51	49
212	IP 19405	Chad	NO	56	10	59	57
			MEAN	56	9	54	49
			Mean exced	97	85	102	90

Supplimental Table S3. Selected 39 accessions with country of origin, field performance and mineral nutrients (ICP data) across two crop seasons

S. No.	Accession identifier	Country of origin	Part of Mini Core	Mean of Summer 2011 and Rainy 2011 seasons													
				**Bloom** (days)	**TSW** (g/ 1000 grains)	**Fe** (mg kg^{-1})	**Zn** (mg kg^{-1})	**Mn** (mg kg^{-1})	**Cu** (mg kg^{-1})	**Mo** (mg kg^{-1})	**Ni** (mg kg^{-1})	**Ca** (mg kg^{-1})	**Mg** (mg kg^{-1})	**Na** (mg kg^{-1})	**K** (mg kg^{-1})	**P** (mg kg^{-1})	**S** (mg kg^{-1})
1	IP 3329	India	YES	60	7	78	62	14	7.3	1.9	1.4	155	1438	14	4800	4400	1533
2	IP 3626	India	YES	52	9	74	56	11	6.6	1.5	1.2	161	1453	10	3767	3800	1480
3	IP 3749	India	NO	58	9	68	61	12	5.2	1.9	1.0	140	1335	13	3433	3550	1453
4	IP 4454	India	NO	56	8	70	58	12	5.1	1.5	1.4	85	1420	11	3375	3400	1530
5	IP 5316	Niger	NO	57	11	66	50	15	5.1	1.0	1.1	201	1240	8	4400	3667	1413
6	IP 7208	India	NO	53	7	70	53	15	6.4	1.5	1.7	162	1627	15	4100	4100	1460
7	IP 7536	India	NO	58	7	78	56	17	5.9	1.2	0.8	204	1523	13	4367	4200	1577
8	IP 7838	India	NO	52	9	67	56	12	5.9	1.4	1.4	196	1373	11	3800	3600	1543
9	IP 8972	Togo	NO	46	11	93	47	16	5.9	1.0	1.9	180	1377	16	4433	3700	1303
10	IP 9301	Togo	NO	49	14	83	55	13	5.9	1.0	1.8	192	1220	13	4400	3433	1253
11	IP 9351	Ghana	NO	46	14	69	49	14	6.9	0.6	1.1	229	1127	13	3500	3125	1303
12	IP 9407	Ghana	NO	45	13	62	43	14	5.4	1.3	0.9	201	1243	14	4233	3467	1280
13	IP 9416	Ghana	NO	45	14	75	55	15	5.7	1.3	0.9	240	1257	13	4050	3450	1255
14	IP 9496	Ghana	NO	42	15	68	44	13	5.1	1.0	1.4	155	1133	20	3875	3150	1243
15	IP 9572	Ghana	NO	64	10	52	46	16	6.1	1.3	1.5	164	1740	20	5133	4733	1703
16	IP 10394	India	NO	51	14	88	52	16	6.5	1.1	1.3	201	1263	15	3900	3350	1300
17	IP 10471	Zimbabwe	NO	61	9	72	61	12	5.4	1.8	0.9	105	1390	12	4967	4033	1455
18	IP 11316	Burkina Faso	NO	56	16	67	57	13	4.1	1.7	0.7	150	1213	14	4600	3575	1183
19	IP 11320	Burkina Faso	NO	47	15	50	40	12	4.0	1.2	0.9	148	1130	16	4267	3200	1123
20	IP 11353	Burkina Faso	NO	67	12	53	48	11	5.9	1.7	1.2	162	1318	12	4500	3675	1355
21	IP 11584	Burkina Faso	NO	60	11	76	65	14	5.5	2.2	1.4	208	1333	16	4825	3650	1353
22	IP 11784	India	NO	51	10	71	62	12	4.6	1.7	0.9	160	1147	13	3167	2900	1467
23	IP 12181	Nigeria	NO	63	9	76	64	14	5.0	0.9	1.1	176	1293	10	3975	3550	1438
24	IP 12507	India	NO	51	10	76	42	12	6.7	1.5	1.2	128	1405	15	4775	4100	1583
25	IP 12682	India	NO	54	11	82	57	10	7.2	1.5	0.7	119	1317	13	4900	4033	1237
26	IP 12939	Ghana	NO	43	13	66	55	13	6.0	1.0	0.9	159	1213	14	3825	3375	1245
27	IP 13384	Uganda	NO	55	11	48	33	17	7.1	1.2	0.8	91	1275	9	3400	3500	1268
28	IP 13900	Burkina Faso	NO	51	12	63	58	13	6.3	1.7	1.4	138	1223	15	3650	3433	1278
29	IP 14148	Zimbabwe	NO	59	10	69	55	12	5.3	1.2	0.9	205	1307	13	4667	4100	1298
30	IP 15402	India	NO	54	8	72	45	16	5.7	1.4	1.2	249	1343	13	4125	3550	1323
31	IP 15614	Burkina Faso	NO	59	6	71	61	17	7.5	1.8	1.1	229	1837	22	4633	4500	1523
32	IP 15817	Tanzania	NO	60	10	78	48	14	5.8	1.1	1.2	138	1380	17	4350	3325	1220
33	IP 17217	Burkina Faso	NO	59	9	64	59	16	5.6	2.2	1.3	179	1613	14	4400	4025	1638
34	IP 17554	Togo	NO	44	13	53	33	14	3.7	0.9	1.0	140	1207	14	4033	3067	1150
35	IP 17566	Togo	NO	41	14	68	50	13	5.9	1.2	1.4	167	1230	15	3775	3325	1305
36	IP 17620	Togo	NO	53	13	91	57	16	3.9	0.7	1.3	212	1327	18	3867	3300	1195
37	IP 17690	Togo	NO	53	14	78	53	11	5.6	1.5	1.1	189	1167	10	3933	3400	1293
38	IP 17707	Togo	NO	53	12	82	51	17	6.1	1.3	1.1	235	1280	18	4400	3650	1400
39	IP 17878	India	NO	50	13	70	56	14	6.3	1.1	1.2	175	1358	13	4200	3750	1358
			Mean	53	11	71	53	14	6	1	1	173	1335	14	4174	3645	1367

Supplimental Table S4. Clustering of 212 core collection accessions based on evaluation in the two seasons, ICRISAT Center, Patancherı

Clusters	ıber of acces	IP Nos		50% flower	TSW (g)	Fe (ppm)	Zn
Cluster-1	32	3106 3296 3626 3749 3799 4197 4454 5070 5316 6584 7208 7734 7838 8426 9286 10811 11584 11784 11893 12682 12768 13191 14210 15402 15817 16617 16770 17028 17064 18389 18910 19405	Min	50	7	50	47
			Max	66	13	83	66
			Mean	56	10	65	55
				bc	c	a	b
			SE	0.59	0.24	1.27	1.01
Cluster-2	42	3122 3138 3183 3284 3890 4466 4695 4974 5272 5695 7377 7440 7762 8074 8409 8562 8593 9618 10543 10694 10761 10820 11049 11358 11391 11537 13154 14028 14942 15159 15372 15872 16380 18500 18657 18702 19067 19072 19078 19141 19263 19344	Min	48	5	34	30
			Max	63	11	50	49
			Mean	56	8	42	40
				bc	de	d	d
			SE	0.64	0.19	0.63	0.71
Cluster-3	26	3163 4759 4779 4828 4979 5131 5222 6013 6639 6793 7020 7660 8867 11353 12967 13180 13384 13971 14026 14148 14991 15177 15273 15297 18281 19175	Min	47	7	36	42
			Max	67	13	68	54
			Mean	54	10	53	47
				c	cd	bc	c
			SE	0.8	0.34	1.24	0.5
Cluster-4	7	3329 4747 6869 10964 15092 17217 18246	Min	56	7	57	61
			Max	61	9	75	74
			Mean	59	8	68	68
				ab	de	a	a
			SE	0.74	0.34	2.15	1.66
Cluster-5	32	3432 4542 5056 7108 7118 7470 7536 8344 8429 8564 9140 9426 9678 9969 10471 11677 12138 12181 12627 13565 14362 15218 15234 15257 15614 15899 16911 18421 18786 18797 19215 19388	Min	50	6	44	47
			Max	70	10	72	65
			Mean	59	8	57	55
				ab	e	b	b
			SE	0.77	0.2	1.04	0.85
Cluster-6	29	3865 4499 5031 6682 6882 6897 8069 8863 9572 9981 10202 10290 12020 12901 13290 14418 15233 15304 16304 16426 17125 18621 18679 18722 18780 19040 19229 19299 19305	Min	55	6	35	40
			Max	69	9	58	56
			Mean	62	8	45	46
				a	e	cd	c
			SE	0.61	0.16	0.91	0.7
Cluster-7	23	3999 4120 4291 4962 5045 5666 6530 7633 8208 8339 9840 11320 11875 12507 12805 13137 14778 15423 15498 16197 17554 17632 18742	Min	41	8	38	34
			Max	55	15	65	48
			Mean	50	11	50	42
				d	b	bcd	cd
			SE	0.76	0.35	1.41	0.67
Cluster-8	21	6745 8972 9301 9351 9407 9416 9496 10394 11316 12925 12939 13900 15917 16449 17566 17620 17690 17707 17878 17978 19190	Min	41	11	56	46
			Max	56	15	90	59
			Mean	48	13	72	52
				d	a	a	b
			SE	0.95	0.29	2.17	0.92

Cluster means with similar alphabets not differ significant for that trait as per SNK test

SE- Standard error

Supplimental Table S5. Clustering of 39 core collection accessions based on evaluation in the two seasons, ICRISAT Center, Patancheru, India.

Cluster	# Geno	IP Nos	Promising IP Nos		Bloom (d)	TSW	(Fe	Zn	Mn	Cu	Mo	Ni	Ca	Mg	Na	K	P	S
Cluster 1	7	3329 7208 7536 9572 12507 15614 17217	3329, 15614, 7536 and 17217	Min	51	6	52	42	12	5.6	1.21	0.84	128	1405	13	4100	4025	1460
				Max	64	10	78	62	17	7.5	2.17	1.67	229	1837	22	5133	4733	1703
				Mean	58	8	70	54	15	6.5	1.64	1.29	175	1597	16	4601	4294	1574
					ab	c	b	a	a	a	a	a	ab	a	a	a	a	a
				SE	1.7	0.6	3.6	2.9	0.6	0.26	0.13	0.1	13	59	1.3	129	98	30
Cluster 2	11	3626 3749 4454 5316 7838 11784 12181 13900 15817 17690 17878	Low-Fe genotypes	Min	50	8	63	48	11	4.63	0.89	0.88	85	1147	8	3167	2900	1220
				Max	63	14	78	64	15	6.61	1.95	1.4	201	1453	17	4400	3800	1543
				Mean	55	10	71	56	13	5.59	1.41	1.18	160	1308	12	3823	3489	1407
					ab	b	b	a	b	a	ab	a	b	b	a	b	bc	b
				SE	1.3	0.6	1.5	1.5	0.4	0.2	0.1	0.05	10	31	0.8	121	74	32
Cluster 3	7	8972 9301 9416 10394 15402 17620 17707	8972, 15402, 17707 and 17620	Min	45	8	72	45	13	3.91	0.72	0.91	180	1220	13	3867	3300	1195
				Max	54	14	93	57	17	6.49	1.44	1.91	249	1377	18	4433	3700	1400
				Mean	50	12	83	52	16	5.67	1.11	1.36	216	1295	15	4168	3490	1290
					bc	ab	a	a	a	a	b	a	a	b	a	ab	bc	bc
				SE	1.3	0.8	3	1.6	0.5	0.31	0.09	0.14	10	21	0.9	92	56	24
Cluster 4	8	9351 9407 9496 11320 12939 13384 17554 17566	9351, 17566, 9496 and 9407	Min	41	11	48	33	12	3.68	0.61	0.75	91	1127	9	3400	3067	1123
				Max	55	15	69	55	17	7.05	1.34	1.44	229	1275	20	4267	3500	1305
				Mean	45	14	61	43	14	5.51	1.04	1.04	161	1195	14	3864	3276	1239
					c	a	b	b	ab	a	b	a	b	b	a	b	c	c
				SE	1.5	0.5	3.1	2.8	0.5	0.44	0.08	0.09	15	20	1	111	58	24
Cluster 5	6	10471 11316 11353 11584 12682 14148	12682, 11584, 10471 and 14148	Min	54	9	53	48	10	4.14	1.21	0.68	105	1213	12	4500	3575	1183
				Max	67	16	82	65	14	7.16	2.23	1.44	208	1390	16	4967	4100	1455
				Mean	59	12	70	57	12	5.58	1.7	0.98	158	1313	13	4743	3844	1313
					a	ab	b	a	b	a	a	a	b	b	a	a	b	bc
				SE	1.8	1	4	2.3	0.6	0.4	0.14	0.12	17	23	0.6	75	96	39

Cluster means with similar alphabets not differ significant for that trait
